# Phylogenomics Yields New Insight Into Relationships Within Vernonieae (Asteraceae)

**DOI:** 10.3389/fpls.2019.01224

**Published:** 2019-10-17

**Authors:** Carolina M. Siniscalchi, Benoit Loeuille, Vicki A. Funk, Jennifer R. Mandel, José R. Pirani

**Affiliations:** ^1^The Mandel Lab, Department of Biological Sciences, University of Memphis, Memphis, TN, United States; ^2^Laboratório de Sistemática Vegetal, Departamento de Botânica, Instituto de Biociências, Universidade de São Paulo, São Paulo, Brazil; ^3^Departamento de Botânica - CCB, Universidade Federal de Pernambuco, Recife, Brazil; ^4^Department of Botany, National Museum of Natural History, Smithsonian Institution, Washington, DC, United States

**Keywords:** Compositae, Hyb-Seq, phylogeny, target capture, *Vernonia*

## Abstract

Asteraceae, or the sunflower family, is the largest family of flowering plants and is usually considered difficult to work with, not only due to its size, but also because of the abundant cases of polyploidy and ancient whole-genome duplications. Traditional molecular systematics studies were often impaired by the low levels of variation found in chloroplast markers and the high paralogy of traditional nuclear markers like ITS. Next-generation sequencing and novel phylogenomics methods, such as target capture and Hyb-Seq, have provided new ways of studying the phylogeny of the family with great success. While the resolution of the backbone of the family is in progress with some results already published, smaller studies focusing on internal clades of the phylogeny are important to increase sampling and allow morphological, biogeography, and diversification analyses, as well as serving as basis to test the current infrafamilial classification. Vernonieae is one of the largest tribes in the family, accounting for approximately 1,500 species. From the 1970s to the 1990s, the tribe went through several reappraisals, mainly due to the splitting of the mega genus *Vernonia* into several smaller segregates. Only three phylogenetic studies focusing on the Vernonieae have been published to date, both using a few molecular markers, overall presenting low resolution and support in deepest nodes, and presenting conflicting topologies when compared. In this study, we present the first attempt at studying the phylogeny of Vernonieae using phylogenomics. Even though our sampling includes only around 4% of the diversity of the tribe, we achieved complete resolution of the phylogeny with high support recovering approximately 700 nuclear markers obtained through target capture. We also analyzed the effect of missing data using two different matrices with different number of markers and the difference between concatenated and gene tree analysis.

## Introduction

The Asteraceae, or Sunflower family, comprise about 10% of the diversity of angiosperms and are widespread occurring in almost all biomes and environments. Some groups comprise major components in threatened ecosystems, like the tribes Lychnophorinae in the Brazilian *campos rupestres* and Corymbieae and Arctotideae in the South African *fynbos* (Karis et al., 2009; Loeuille et al., 2019). More than 40 species have been domesticated, e.g., lettuce, artichoke, sunflower, safflower, stevia, and chicory, and some noxious weeds also belong in the family, e.g., *Mikania micrantha *Kunth., *Chromolaena odorata* (L.) R.M.King & H.Rob., and *Ambrosia artemisiifolia* L. (ragweed).

Although the systematics of the family has been studied since before the Linnean system (e.g., de Tournefort, 1700; Vaillant, 1719-1723), and the most used infrafamiliar classification has remained largely unchanged since its publication (Cassini, 1819), our understanding of the phylogenetic relationships within the family has drastically changed in the last decades. Morphological and molecular phylogenies challenged the long-standing view that the Heliantheae alliance was the earliest diverging tribe in the family, showing that they are actually highly nested within the family (Jansen and Palmer, 1988; Bremer, 1994; Funk et al., 2009).

Nevertheless, tackling the backbone phylogeny of the family has always been challenging, as there is a well-documented evidence of an abundance of polyploidy, hybridization events, ancient whole-genome duplications, and explosive radiations (Barker et al., 2008; Semple and Watanabe, 2009; Barker et al., 2016). In the past 5 years, with the availability of second-generation sequencing methods and their adaptation for use with non-model organisms, two different approaches to understanding evolutionary relationships in Asteraceae have emerged. The first is the use of a set of RNA or DNA probes that target specific orthologous loci within the genome, allowing them to be captured, enriched, and sequenced (Mandel et al., 2014), and the second is the use of transcriptome sequencing to acquire orthologous loci (Huang et al., 2016), with both being used to produce family-level phylogenies (Huang et al., 2016; Mandel et al., 2017; Mandel et al., 2019).

While transcriptome sequencing is straightforward in relation to sample processing and wet lab procedures, the main drawbacks are the need to collect samples in a way that preserves the RNA in the tissue, which precludes using herbarium specimens as sources for sampling, and the fact that gene expression is variable, which may impact locus recovery across samples and making the possibility of combining data from different studies challenging (Wen et al., 2015).

Target capture associated with genome skimming arose initially as a way to obtain sequences from ultraconserved elements in the genome of vertebrates and invertebrates (Cronn et al., 2012; Faircloth et al., 2012; Grover et al., 2012) but has been further extended into plant phylogenomics recently, with the release of lower or higher taxonomic level probes, such as for family Asteraceae (Mandel et al., 2014), genera *Protea* (Mitchell et al., 2017), *Heuchera* (Folk et al., 2015), and *Inga* (Nicholls et al., 2015) and, more recently, for all angiosperms (Johnson et al., 2019). Although sample preparation requires extra steps, time, and additional cost from the target capture kit, target recovery is usually consistent within a lineage and allows the combination of data generated across different studies. Given the possibility of using previously collected material for DNA extraction, such as from herbarium collections, samples preserved in silica gel, or DNA banks, target capture is appealing in the context of the increasing challenges of securing financial and human resources for field work.

The Asteraceae conserved ortholog set (COS) kit developed by Mandel et al. (2014) has been successfully tested across the family (Mandel et al., 2017; Mandel et al., 2019) and within higher-nested lineages (Herrando-Moraira and The Cardueae Radiations Group, 2018). Aiming to study the effectiveness of this method in a lineage known for its complicated evolutionary and taxonomic history, we generated a phylogeny of tribe Vernonieae.

**Figure 1:**
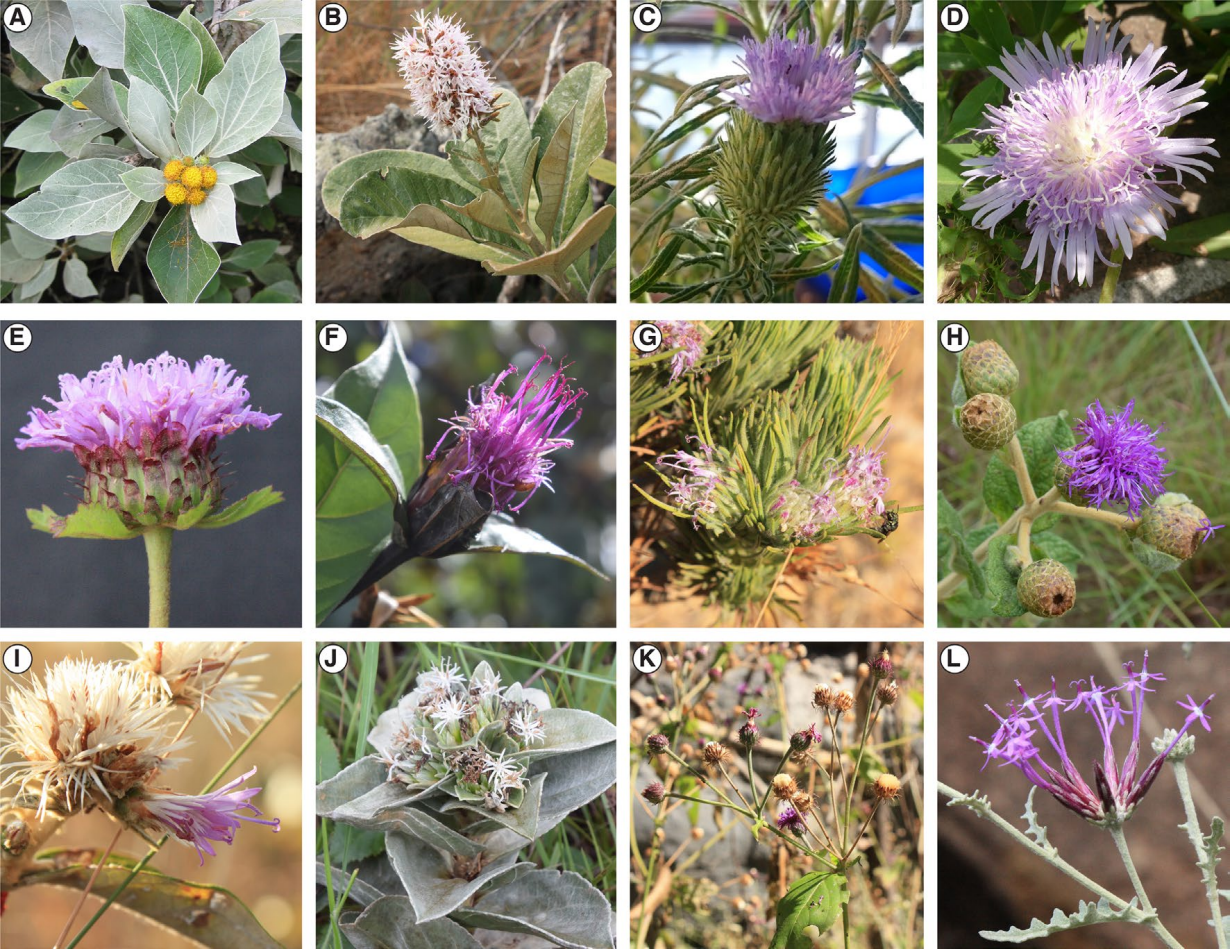
Diversity in Vernonieae and related taxa. **(A)** *Distephanus populifolius* (Distephaninae). **(B)** *Moquinia racemosa* (tribe Moquinieae), **(C)** *Centrapalus pauciflorus* (Centrapalinae), **(D)** *Stokesia laevis* (Stokesiinae), **(E)** *Centratherum punctatum *(Lychnophorinae), **(F)** *Hololepis pedunculata* (Lychnophorinae), **(G)** *Lychnophora ericoides* (Lychnophorinae), **(H)** *Lessingianthus monocephalus* (Lepidaploinae), **(I)** *Strophopappus speciosus* (Lepidaploinae), **(J)** *Soaresia velutina* (Elephantopinae), **(K)** *Heterocypsela andersonii* (Dypterocypselinae), **(L)** *Chresta hatschbachii* (Chrestinae). Photos by VF **(A)** and CS **(B**–**L)**.

Vernonieae contains about 1,500 species and is distributed in the New and Old World, with the main diversity centers in Africa and South America. Members of Vernonieae are easily recognized by the homogamous heads composed only by tubular florets, the predominance of pinkish-purplish corollas, and the often recurved style branches (**Figure 1**). The circumscription of the tribe has hardly changed since Cassini’s first description (1819), but genera circumscription within it has drastically changed since the 1980s.

Most of the species of the tribe have been previously placed in the comprehensive genus *Vernonia*, with more than 1,000 species. There were several attempts at creating infrageneric classifications for *Vernonia* (Jones, 1979; Jones, 1981; Jeffrey, 1988), which culminated in its pulverization into several other genera (Robinson, 1999a; Robinson, 1999b), such as *Centrapalus*, *Cyrtocymura*, *Distephanus*, *Lepidaploa*, *Lessingianthus*, and *Vernonanthura*.

**Figure 2:**
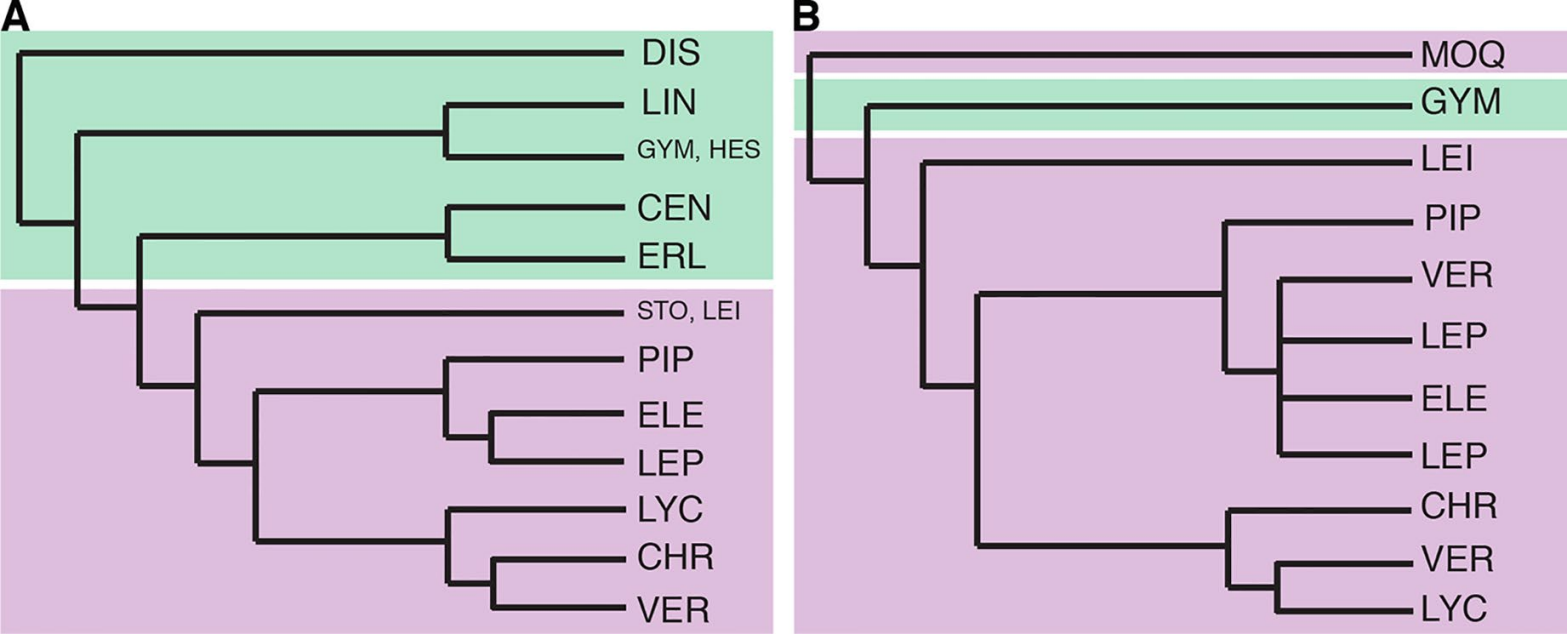
Previous Vernonieae phylogenies. **(A)** redrawn from **Figure 2** in Keeley et al. (2007), Bayesian analysis of the combined molecular dataset. **(B)** redrawn from **Figure 2** in Loeuille et al. (2015a), strict consensus of 96 equally most parsimonious trees based on the combined molecular data. Branch lengths are illustrative, without real value. Green shading represents taxa with mostly Old World distribution and purple shading those with mostly New World distribution. CEN, Centrapalinae; CHR, Chrestinae; DIS, Distephaninae; ELE, Elephantopinae; ERL, Erlangeinae; GYM, Gymnantheminae; HES, Hesperomanniinae; MOQ, Moquinieae; LEI, Leiboldiinae; LEP, Lepidaploinae; LIN, Linziinae; LYC, Lychnophorinae; PIP, Piptocarphinae; STO, Stokesiinae; VER, Vernoniinae.

The first phylogenetic studies in Vernonieae focused on the relationships within *Vernonia* (Keeley and Turner, 1990) and showed that the African species of the genus form a grade leading to a more speciose clade of New World species. In addition, their work demonstrated that the species now included in *Distephanus* (**Figure 1A**), a genus from Madagascar and South Africa, were the sister to the whole genus. Keeley et al. (2007) expanded this first phylogeny, using two chloroplast regions (*ndh*F and *trn*L-F) and ribosomal ITS, and focused on the whole tribe, already including several of the taxonomic changes that occurred since 1990 (**Figure 2A**). Again, the division between Old World and New World groups is clear, as well as the outgroup position of *Distephanus*. The complexity of the relationships in the New World clade also becomes evident with several instances where members of clades are found in distant locations, such as the clade formed by *Vernonia* s.str., found in North America that is a sister to a clade formed by genera from Central America and Brazil, which in turn is a sister to a large clade of Brazilian species. *Stokesia* (**Figure 1D**), a monotypic genus from the Southeastern USA and the only species in the tribe to present zygomorphic florets, seems somewhat problematic, with its position varying depending on the markers used but generally emerges close to Leiboldiinae, in the transition between the larger Old and New World clades in combined analyses.

In 2015, Loeuille et al. published an in-depth phylogeny of the American Vernonieae (**Figure 2B**), focusing on the evolution of secondary heads on the group, using internal transcribed spacer, two chloroplast regions (*ndh*F and *trn*L-F), and a morphological matrix. The division between Old and New World was also found, but *Distephanus* was not sampled. In this work, it was clear that some of the new subtribes and even new genera proposed in the years before were not monophyletic, such as subtribe Vernoniinae, whose members are spread out in several clades or in genus *Lessingianthus*. The relative position of each clade also is different from that found in Keeley et al. (2007), especially with regard to the clades containing subtribes Lychnophorinae, Chrestinae, and *Vernonia* s.str. These relationships also vary depending on the dataset used, and the position of *Stokesia* also changes depending on the analysis, with it emerging with low support as a sister to subtribe Chrestinae, well within the New World clade and not in a transitional position, or as a sister to Leiboldiinae, as seen in Keeley et al. (2007).

Regarding the position of Vernonieae within Asteraceae, the tribe has usually been placed in Cichorioideae and is known to be closely related to Liabeae (Keeley et al., 2007; Panero and Funk, 2008). Relationships within Cichorioideae have always been unstable (Funk and Chan, 2009), with recent evidence that tribe Cichorieae might be more closely related to subfamily Asteroideae than to the rest of the tribes in Cichorioideae itself (Mandel et al., 2017; Mandel et al., 2019). In the megatree by Funk et al. (2009), the small South American tribe Moquinieae (**Figure 1B**) emerges in a polytomy with Vernonieae and *Distephanus* and also presents alternative placements in relation to both in an in-depth analysis of the relative positions of the tribes in Cichorieae (Funk and Chan, 2009), showing these relationships require further investigation.

Based on the hitherto known information about the phylogeny of Vernonieae and focusing on resolving some of the controversies between previous works, we carried out a phylogenetic study employing genomic methods, in order to: 1) understand the relationships among different subtribes in Vernonieae, especially among South American groups, 2) define the relationships among Moquinieae, *Distephanus*, and the core Vernonieae, and 3) understand the impact of different levels of missing data and of concatenated and pseudo-coalescence methods in the phylogenetic analysis.

## Material and Methods

### Outgroup Choice and Taxon Sampling

As Liabeae, Moquinieae, and *Distephanus* have been shown to be the sister groups to Vernonieae in previous works (Keeley and Robinson, 2009; Loeuille et al., 2015a), we chose as outgroup one taxon from Liabeae, *Munnozia gigantea*, and sampled as ingroup the only two representatives from tribe Moquinieae (*Moquinia racemosa* and *Pseudostifftia kingii*), *Distephanus ambonguensis*, and another 56 species representing 29 different genera from Vernonieae (4% of the species ascribed to the tribe). Taxa from 12 subtribes (from the 21 defined by Keeley and Robinson, 2009) were included, of which nine occur in South America and three are distributed in Africa/Asia. The sampling was focused on the three large South American clades that showed uncertain relationships based on previous studies (Keeley et al., 2007, Loeuille et al., 2015a). Sequences for 25 taxa were newly generated for this study, while sequences for the remaining 35 species were previously published elsewhere (Mandel et al., 2014; Mitchell et al., 2017; Mandel et al., 2019). A list of sampled species, herbarium vouchers, and publication status is presented in **Supplemental Material Table 1**.

### DNA Extraction and Sequencing

Leaf samples were collected from live plants in the field and preserved in silica gel or extracted from herbarium sheets. Dried leaves were ground using a GenoGrinder 3000 (Spex® Sample Prep), and total DNA was extracted using E.Z.N.A.® SQ Plant DNA Kit from Omega Biotek, with addition of polyvinylpyrrolidone and ascorbic acid to the first extraction buffer (10-ml SQ1 buffer, 100-mg polyvinylpyrrolidone, 90-mg ascorbic acid). When necessary, the extracted DNA was cleaned with the E.Z.N.A.® Cycle Pure Kit from Omega Biotek to increase purity. Extracted samples were quantified using fluorometry (Qubit 3.0, ThermoFisher Scientific), diluted as necessary, and sheared to a target size of 400–500 bp using a sonicator (Covaris S series or QSonica Q500). DNA fragmentation was verified through electrophoresis in 1% agarose gels.

Libraries were prepared with the NEBNext Ultra II DNA Library Prep Kit for Illumina (New England Biolabs Inc.) with an initial concentration of at least 500 ng of total DNA, according to the manufacturer’s instructions, using 15 cycles on the last amplification step. Final library concentrations and sizes were checked using Qubit and gel electrophoresis. Libraries were pooled in groups of four in equimolar concentration, containing 125 ng of each library, and target capture was performed using the MYbaits COS: Compositae/Asteraceae 1kv1 kit (Arbor Biosciences), using a 36-h incubation time and 15 cycles on the last amplification step. Details on the targets and method can be found in Mandel et al. (2014).

Quality checking with a Bioanalyzer instrument and sequencing were carried out at Macrogen Inc. (South Korea), in an Illumina HiSeq2500 device, in paired-end, high-throughput mode.

### Sequence Assembly and Mapping

Trimming of Illumina adaptors was carried out using Trimmomatic (Bolger et al., 2014), and reads were assembled into contigs using SPAdes (Bankevich et al., 2012), with kmer lengths of 21, 33, 55, 77, 99, and 127. The sequences were matched back to the original probes using the phyluce pipeline (Faircloth, 2016), which generated individual alignments for each one of the original targeted regions. These alignments were then concatenated to generate two different matrices for phylogenetic analysis, using the “phyluce_align_get_only_loci_with_min_taxa” script within the phyluce pipeline, specifying different degrees of completeness in relation to number of loci contained in the final matrix. One matrix contains all loci recovered for all taxa (herewith called total matrix), and the other contains only loci that were recovered for at least 75% of the taxa (called 75% matrix). This approach was chosen to study the effect that different levels of missing data would have over tree topology and statistical support. General information about the matrices was obtained using AMAS (Borowiec, 2016) and files generated by the phyluce pipeline.

### Phylogenetic Analysis

All analyses described were carried out with both datasets, total and 75%, containing invariable characters, using *M. gigantea* as outgroup. The resulting trees are referred to as “total tree” and “75% tree” throughout the results and discussion. Molecular evolution models were evaluated in jModelTest2 (Guindon and Gascuel, 2003; Darriba et al., 2012), using the corrected Akaike Information Criterion and Bayesian Information Criterion to choose between models. The chosen model was GTR + I + G for both matrices and both information criteria. Maximum likelihood (ML) analyses were run on RAxML (Stamatakis, 2014) in the rapid bootstrapping mode, always using 1,000 bootstraps and 25 threads.

The multispecies pseudocoalescence model was evaluated in ASTRAL III (Zhang et al., 2018), using unrooted gene trees generated from the individual locus matrices. Individual evolution models for each gene matrix were obtained with PartitionFinder v.1.1.0, in the RAxML version with rcluster search option and Akaike Information Criterion, with unlinked branch lengths (Stamatakis, 2006; Lanfear et al., 2012; Lanfear et al., 2014). Gene trees were obtained in RAxML, with 100 bootstraps for each matrix. Two different species trees were obtained from the gene trees: one using all recovered loci and other using only loci that were recovered for 75% of the taxa. Branch support was calculated using local posterior probabilities (LPP).

The presence of gene tree conflict and concordance in the pseudocoalescence analyses was checked using PhyParts (Smith et al., 2015). Gene trees used as input for ASTRAL and the resulting species tree generated by the program were unrooted and, thus, had to be rooted to be used as input in PhyParts, which was done using the program pxrr in the package phyx (Brown et al., 2017). Species trees were rooted having *M. gigantea* (from Liabeae) as outgroup. Because the incomplete recovery of loci across taxa leads to several missing taxa in each gene tree, a hierarchical strategy was used to root the gene trees, selecting the outgroup in the following order: *M. gigantea*, *Distephanus ambongensis*, *P. kingii*, *M. racemosa, Vernoniastrum ambiguum*, *Baccharoides anthelmintica*, *Gymnanthemum amygdalinum*, *Centrapalus pauciflorus*, and *Stokesia laevis*. The results from PhyParts were used as input in the phypartspiecharts.py script (Johnson, 2017), to generate a species tree with pie charts in each node showing the proportion of concordant gene trees and conflicting topologies.

The occurrence of long-branch attraction (LBA) was tested using TreeShrink (Mai and Mirarab, 2018), both in the species trees generated by maximum likelihood and pseudocoalescence analyses and in the gene trees used as input to ASTRAL, using a false-positive error rate (α) of 0.05. Pseudocoalescence analyses were rerun with the treated gene trees to account for possible changes in topology and support values.

### Topological Comparison

The topologies obtained with the different analyses and datasets were compared using the adjusted Robinson Foulds distance, as outline in Mitchell et al. (2017) and Herrando-Moraira and The Cardueae Radiations Group (2018). Robinson Foulds distances were calculated in PAUP* v4.0a (Swofford, 2003) for all pairwise comparisons of the six topologies (the total and 75% dataset for each of three analyses: ML, pseudocoalescence, and pseudocoalescence with gene trees treated with TreeShrink analyses) and then manually adjusted using RFadj = RF/(2n - 6), where *n* is the number of nodes in the tree. RFadj ranges from 0 (same topology) to 1 (completely discordant topology). The multidimensional scaling approach implemented in R was used to visualize all the trees in the same treespace, based on the RFadj values, using the function “cmdscale” in the package “stats.”

## Results

### Overview and General Trends

The sequencing generated approximately 902 million reads and approximately 89 billion nucleotides (4 million to 33 million reads per sample). The total matrix contains 61 taxa and has an extension of 729,969 characters, including 707 of the markers contained in the probe set, with 74.9% missing data. The 75% matrix has 61 taxa as well, but the matrix length is of 113,347 characters, containing 89 loci and 34.9% missing data. The number of loci recovered for each taxon varied from 79 in *M. racemosa* to 492 in *C. pauciflorus*, with a median of 249 loci. Although there is a drastic reduction in the number of variable and parsimony-informative sites in the 75% matrix compared with the total matrix, proportionally, the 75% matrix has more parsimony-informative sites (19% against 13%). Comprehensive data for the recovered loci and alignments are found in **Table 1** and **Supplemental Material Table 2**. Raw data are deposited at the National Center for Biotechnology Information (NCBI) Sequence Read Archive, under BioProjects PRJNA540287 and PRJNA546287.

**Table 1:** Comparison of the composition of the total and 75% datasets.

	Total matrix	75% matrix
Number of taxa	61	61
Number of recovered loci	707	89
Length of the concatenated matrix	729,969 bp	113,347 bp
Number of variable sites	235,126 bp (32%)	40,784 bp (35%)
Number of parsimony informative sites	101,707 bp (13%)	22,650 bp (19%)
Proportion of missing data in the concatenated matrix	74.6%	35.7%
Proportion of identical sites in the concatenated matrix	38.5%	22.1%
Average of recovered loci per species (sd; min–max)	247 (87; 79–492)	72 (14; 25–88)
Average number of species recovered per loci (sd; min–max)	20 (15; 3–56)	49 (2; 45–56)
Average sequence length	1,032 (770; 167–10,337)	1,273 (1,117; 372–9,205)

bp, base pairs; sd, standard deviation; min, minimum; max, maximum.

**Figure 3:**
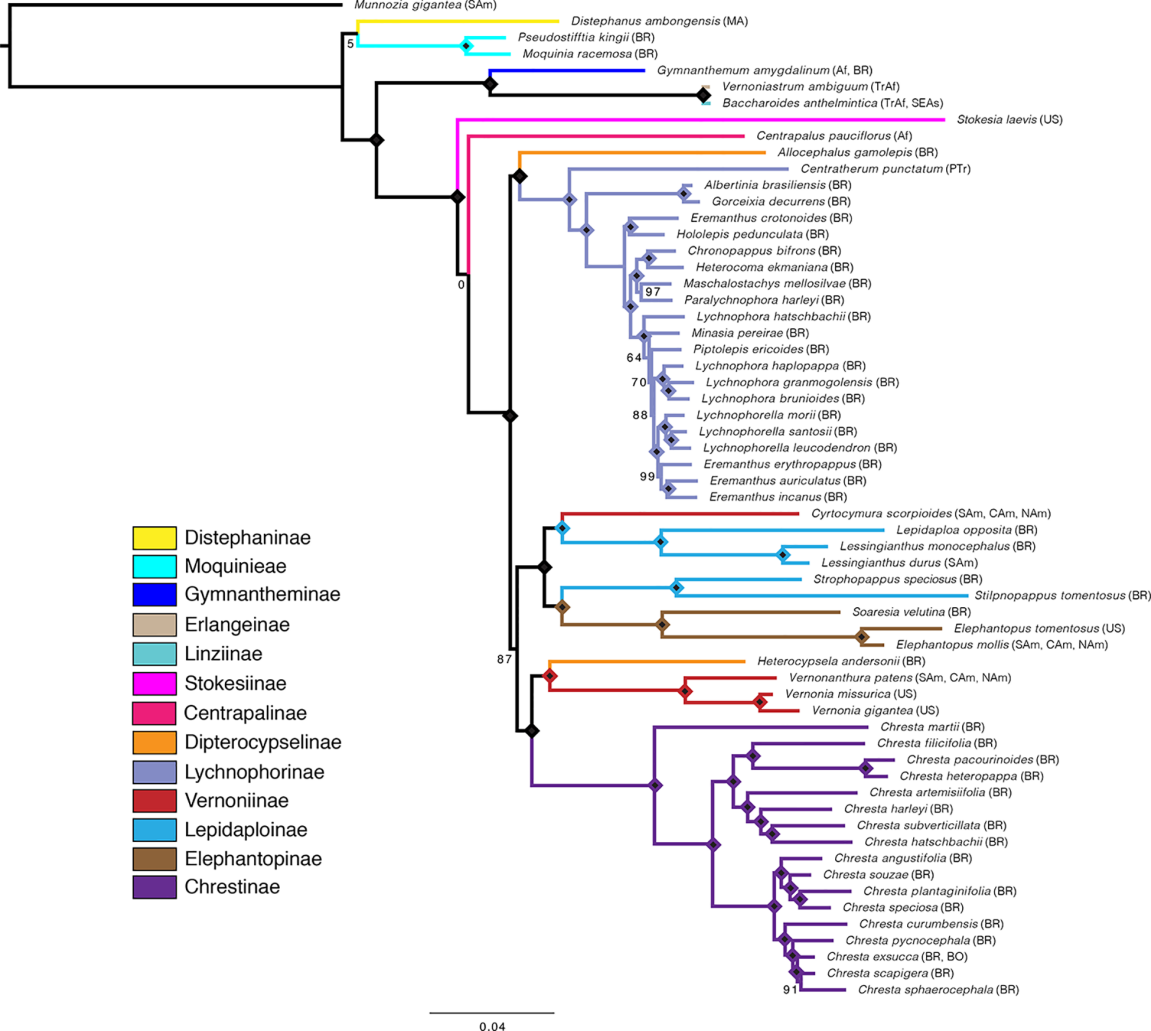
Maximum likelihood tree obtained with the total matrix (707 loci, 729,969 characters), with model GTR + G + I, with 1,000 bootstrap replicates. Diamonds indicate bootstrap value of 100%. Subtribes are coded by color. Geographical distribution indicated in parenthesis: Af, Africa; BO, Bolivia; BR, Brazil; CAm, Central America; MA, Madagascar; NAm, North America; PTr, Pantropical; SAm, South America; SEAs, Southeast Asia; TrAf, Tropical Africa; US, United States of America. Distribution data obtained from Keeley and Robinson (2009), Robinson (1999a, 1999b).

Overall, the four analyses are remarkably consistent, presenting similar topologies and high statistical support (**Figures 2**, **3** and **Supplemental Material Figures 1–3**). Some of the general trends found in all analyses are the position of *Distephanus* and Moquinieae in relation to Vernonieae, these three species form a clade with *Distephanus* as sister group to the other two, although with low support in the ML analysis and high support in the pseudocoalescence (support for total/75% trees: ML bootstrap: 5%, 92%, LPP: 1, 1). Three of the sampled African species form a consistent clade, recovered in all analyses, with *G. amygdalinum* as the sister taxon to *V. ambiguum* and *B. anthelmintica*, with maximum statistical support in all cases. Also, there is an inconsistency in the position of *C. pauciflorus* and *S. laevis* as sister to the South American clade, probably due to the incomplete sampling of African taxa and Mexican subtribe Leiboldiinae. Subtribe Chrestinae, composed only by *Chresta* (**Figure 1L**), is consistently monophyletic with high statistical support (support for total/75% trees: ML bootstrap: 100%, 100%, local PP: 1, 1), and its sister group is a clade formed by *Heterocypsela andersonii* (**Figure 1K**) + *Vernonia s.str. + Vernonanthura*, also with high statistical support (ML bootstrap: 100%, 100%, local PP: 1, 0.99). The relative position of *Chresta exsucca*, *C. scapigera*, and *C. sphaerocephala* varies in the analysis depending on the dataset.

**Figure 4:**
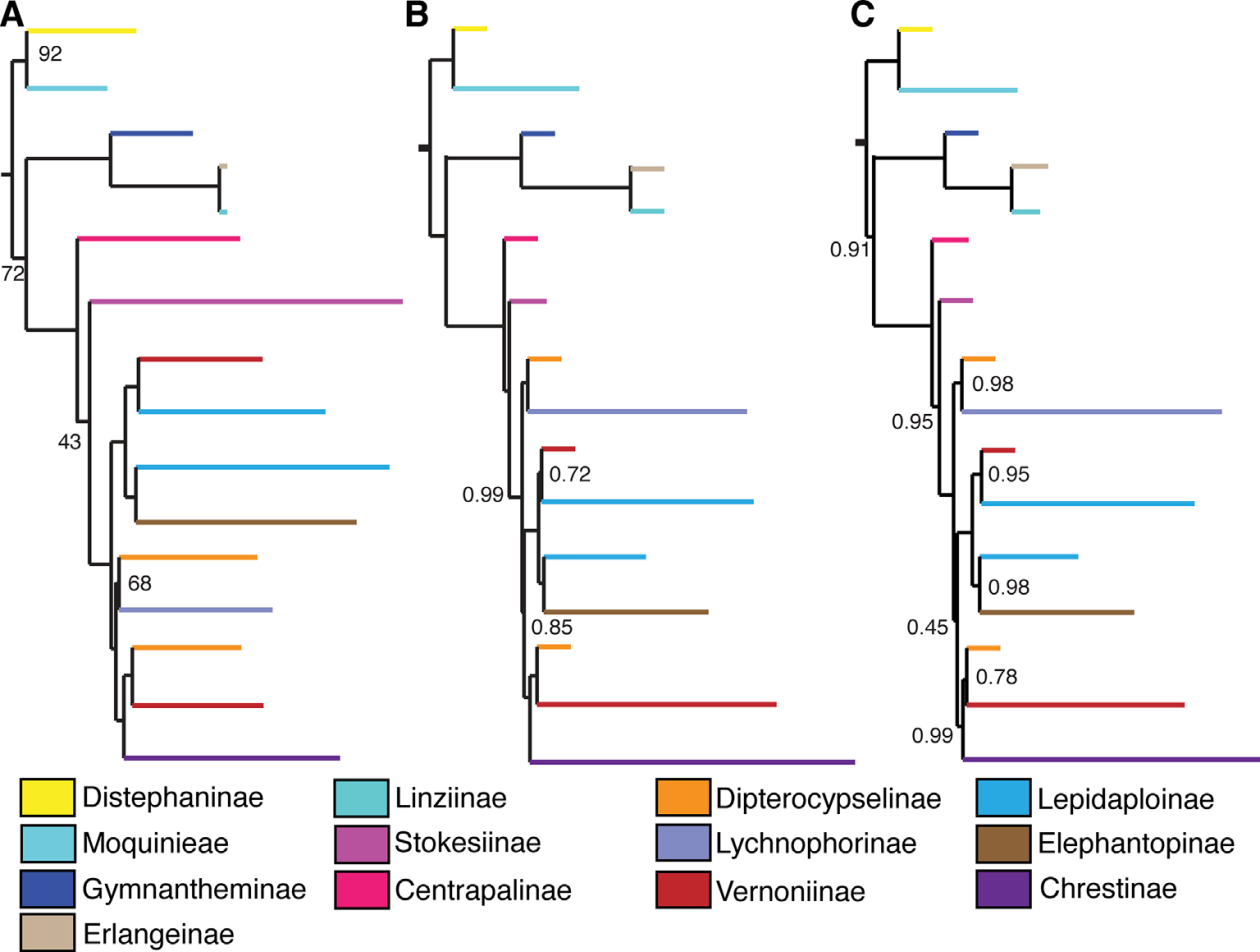
Comparison between backbone trees obtained with different analysis methods and datasets. **(A)** 75% matrix, maximum likelihood. **(B)** Total matrix, pseudocoalescence. **(C)** 75% matrix, pseudocoalescence. Nodes without a value have 100% bootstrap support or local posterior probability of 1. Subtribes are coded by color.

Subtribe Vernoniinae was recovered as non-monophyletic in all analyses; instead, they are split in two clades: *Vernonia* and *Vernonanthura* grouped as the sister clade to Chrestinae and *Cyrtocymura* as sister taxon to Lepidaploinae (**Figures 3** and **4**). Subtribe Lepidaploinae also emerges as non-monophyletic, and although all the species are grouped into a large clade, *Stilpnopappus* and *Strophopappus* (**Figure 1I**) form the sister clade of the Elephantopinae, and *Lepidaploa* and *Lessingianthus* (**Figure 1H**) are in a different clade that also contains *Cyrtocymura*. The monotypic genus *Soaresia* (**Figure 1J**) is included in the Elephantopinae (**Figure 3**).

The two species belonging to subtribe Dipterocypselinae emerge in two distantly related clades, *Heterocypsela* is in a clade with part of the Vernoniinae, and *Allocephalus gamolepis* emerges as sister group of the Lychnophorinae, rendering Dipterocypselinae non-monophyletic.

Lychnophorinae is recovered as monophyletic in all analyses, with some of the relationships within this subtribe being stable, such as the clades formed by *Albertinia brasiliensis* and *Gorceixia decurrens* and *Eremanthus crotonoides* and *Hololepis pedunculata* (**Figure 1F**). There is also a clade formed by four species divided into two clades: *Paralychnophora harleyi + Maschalostachys mellosilvae* and *Chronopappus bifrons + Heterocoma ekmaniana*. The position of *Minasia* and *Piptolepis* in relation to *Lychnophora* and *Eremanthus* varies between the 75% and total datasets, in both the ML and pseudocoalescence analysis.

### Maximum Likelihood

The main difference between the trees obtained in the ML analysis is the relative position of the three large South American clades. In the total ML tree (**Figure 3**), the clade formed by Elephantopinae + Lepidaploinae + Vernoniinae (ELE + LEP + VER) is the sister clade to Chrestinae + Vernoniinae (CHR + VER), and both together form the sister clade of Lychnophorinae (LYC). In the 75% tree (**Supplemental Material Figure 1**), LYC emerges the sister group of CHR + VER, and ELE + LEP + VER is the sister group of the remainder. However, in the 75% tree, these relationships all have total support, while in the total tree, the CHR + VER and ELE + LEP + VER node has 87% of bootstrap support.

The overall support is higher in the total tree, with nine nodes showing support below 100%, while the 75% tree has 13 nodes with lower support. Some nodes with lower support are shared by both trees, such as the basal node in the *Distephanus* + Moquinieae clade, which has lower support in the total tree. Also, the position of *Stokesia* and *Centrapalus* changes in both trees. In the total tree, *Stokesia* emerges before *Centrapalus*, and the node between *Centrapalus* and the South American clade presents no support. In the 75% tree, they are inverted, and the node between *Stokesia* and the South American clade has 43% support.

The number of nodes with lower support within Lychnophorinae and Chrestinae increases in the 75% tree, and there are also changes in topology within these clades, especially in the innermost clade of Lychnophorinae. The analyses with TreeShrink in both trees indicate a possible LBA case with *M. gigantea*, which is the outgroup. Rerunning the analysis with the same α level and removing the outgroup indicate a possible case of LBA with *S. laevis* in the 75% tree, which might explain the inverted position of this taxa and *C. pauciflorus* between the two trees.

### Multispecies Pseudocoalescence

The pseudocoalescence analysis with all loci included 645 gene trees, while the analysis containing only loci that where recovered for at least 75% of the taxa contained 87 gene trees. The normalized quartet score for both datasets was 0.84. Overall, LPP values were strongly affected by reducing the number of loci in the analysis, and the total tree has 12 nodes with support below 1, while the 75% tree has 25 nodes with support below 1.

Differently from the maximum likelihood analyses, there is no variation in the backbone topology between both analyses, with the trees presenting the same relationship among the three large South American clades, where CHR + VER and ELE + LEP + VER are sister clades and LYC is the sister group of this larger clade, in accordance to the topology in the ML total tree. However, the support in the CHR + VER and ELE + LEP + VER node was low in both trees (LPP total/75% tree: 0.85/0.45). There is variation in the topology within clades, especially within Lychnophorinae and in one clade in Chrestinae. There is no variation in the position of *Centrapalus* and *Stokesia*, with *Centrapalus* emerging before *Stokesia*, with high support in both cases (LPP: 1/1, 0.99/0.95) (**Figures 4B, C**, **Supplemental Material Figures 2 and 3**).

Removing taxa that could potentially cause LBA from the gene trees with TreeShrink did not change the topology of the resulting species trees and had confounding effects on overall support. In the total tree (**Supplemental Material Figure 4**), half of the taxa were removed from at least 10 gene trees each, and *B. anthelmintica* and *C. pauciflorus* were removed from 21 and 24 gene trees, respectively (**Supplemental Material Table 1**). The number of nodes with LPP < 1 remained the same (12), but in some of these nodes, the support decreased, such as the node containing CHR + VER and ELE + LEP + VER, in which the support fell from 0.85 to 0.73. In the loci contained in the 75% tree (**Supplemental Material Figure 5**), *M. gigantea* was removed from 10 gene trees and *S. laevis* from 5 gene trees; six other taxa were removed from one tree each (**Supplemental Material Table 3**). The number of nodes with LPP < 1 also remained the same, and the biggest change in support occurred in the *Distaphanus* + Moquinieae node, which fell from 1 to 0.85.

**Figure 5:**
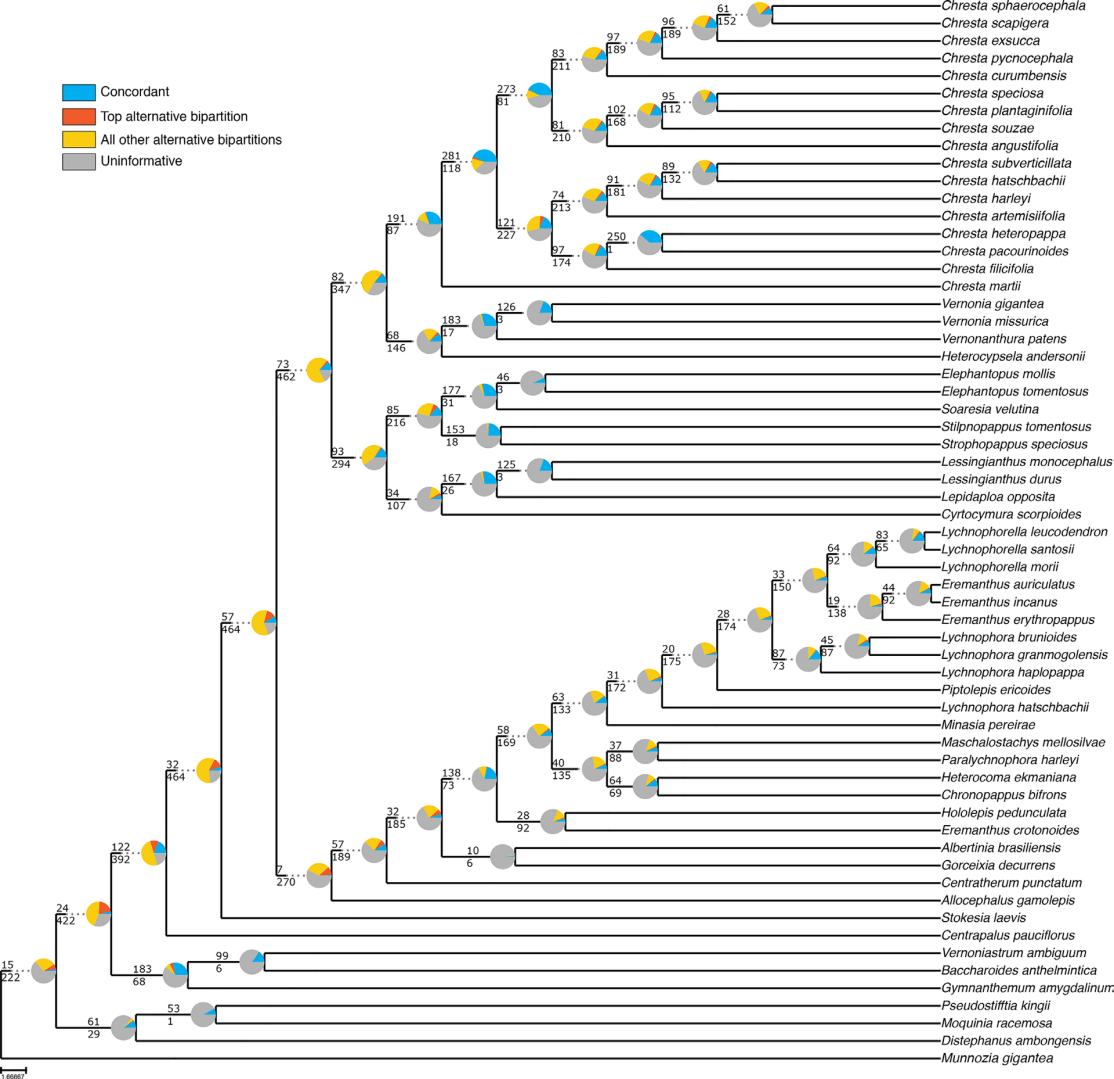
ASTRAL analysis topology of the total dataset showing a summary of concordant and discordant gene trees. For each branch, the top number indicates the number of concordant gene trees and the bottom number the number of conflicting gene trees. The pie charts indicate the proportion of gene trees that support that clade (blue), the proportion that supports the main alternative for that clade (orange), the proportion that supports all other topologies (yellow) or the proportion of uninformative gene trees for that clade (gray).

**Figure 6:**
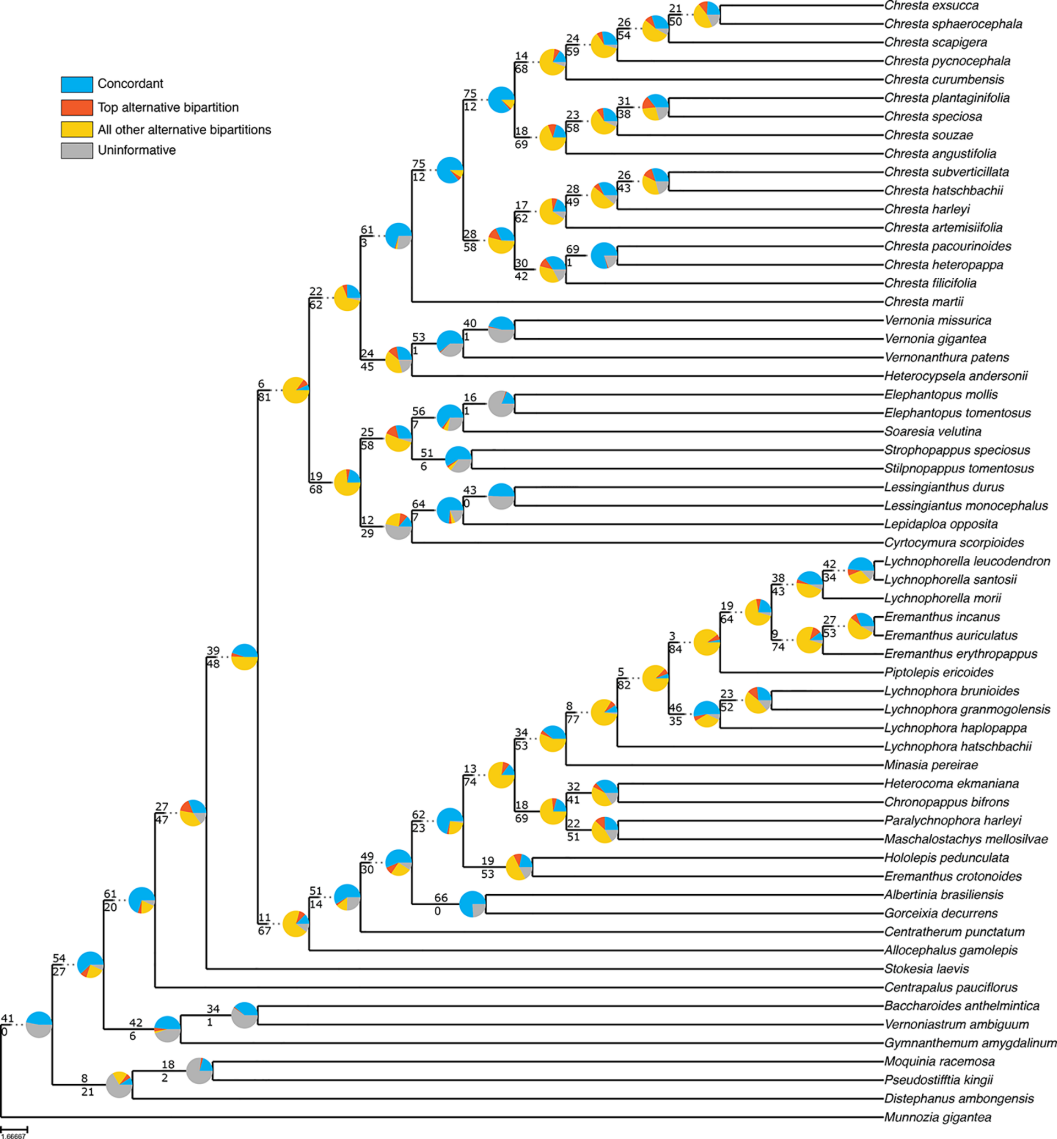
ASTRAL analysis topology of the 75% dataset showing a summary of concordant and discordant gene trees. For each branch, the top number indicates the number of concordant gene trees and the bottom number the number of conflicting gene trees. The pie charts indicate the proportion of gene trees that support that clade (blue), the proportion that supports the main alternative for that clade (orange), the proportion that supports all other topologies (yellow) or the proportion of uninformative gene trees for that clade (gray).

Even though the statistical support generally fell in the 75% tree, the gene tree concordance analysis shows there is less discordance between gene trees than in the total tree. In the total tree, 91% of the nodes show that more than 50% of the gene trees are non-informative for that node, and only small proportions of the trees are concordant (**Figure 5**). The backbone of the tree has lower proportions of non-informative gene trees and also shows concordance with alternative topologies. Nodes within Lychnophorinae are overall more uninformative than in other parts of the tree, also corresponding to the region where support is lower. The 75% tree shows smaller proportions of non-informative gene trees for each node, and 36% of the nodes show a proportion of 50% or more of concordant gene trees (**Figure 6**). The backbone shows higher proportions of concordance, and most of the nodes that showed higher proportions of uninformativeness in the total tree show concordance with alternative topologies in the 75% tree.

### Topological Comparison

**Figure 7:**
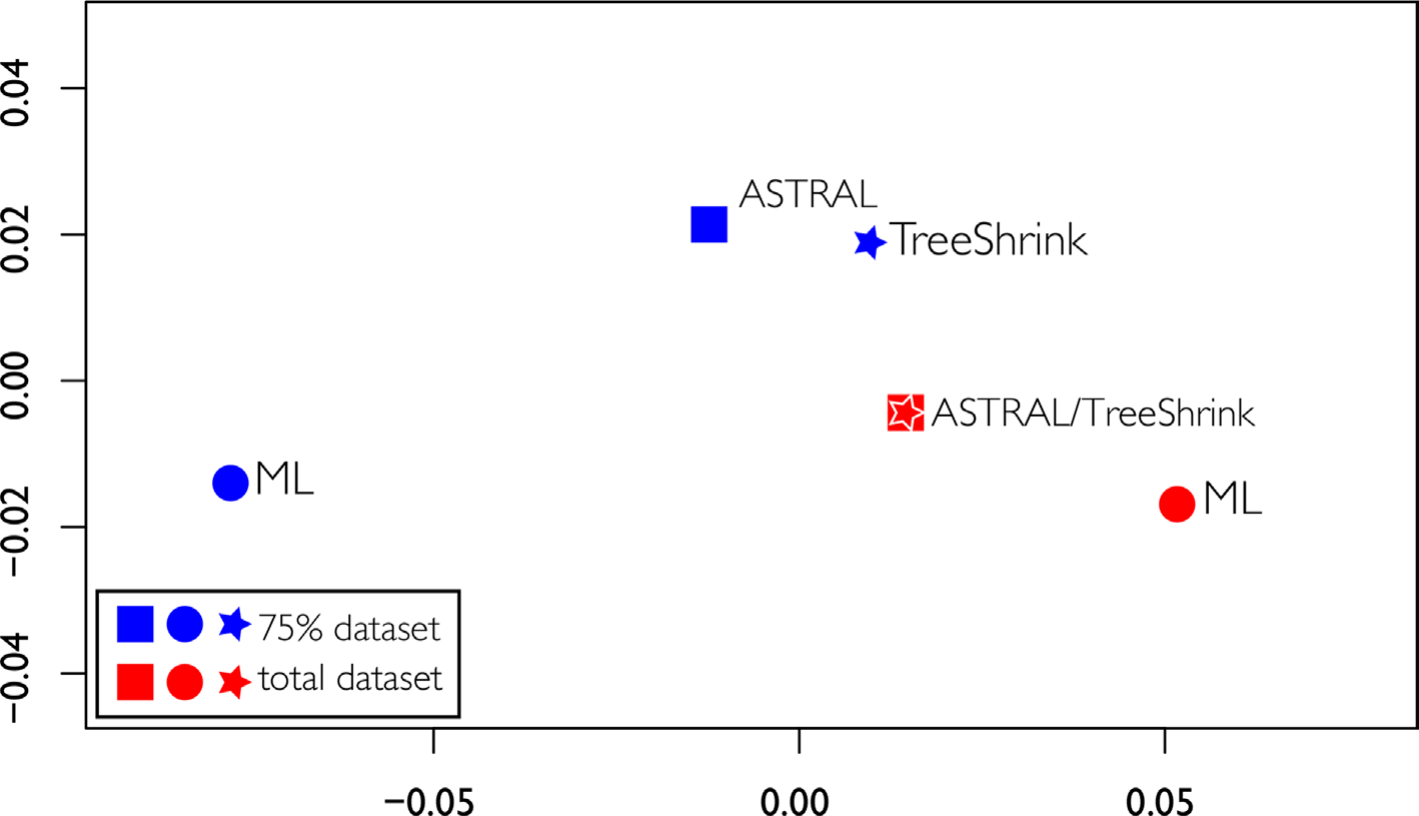
Tree space of the multidimensional scaling of pairwise comparisons of Robinson Foulds distances among the four topologies obtained. ML trees are represented as circles, ASTRAL trees as squares, and ASTRAL trees obtained with gene trees treated with TreeShrink as stars.

Discordant tree topologies were recovered, especially when comparing the two different datasets, including a significant change in the backbone between the two ML topologies. The RFadj values were generally low (**Table 2**), with the largest difference being between the two ML analyses (RFadj = 0.13). The smallest difference was between the total ASTRAL analysis with the total ASTRAL analysis with TreeShrink, which were completely concordant (RFadj = 0.0). Overall the comparisons between the two different datasets had higher discordance, possibly indicating that the dataset, not the analysis method, was driving the differences in topologies, as seen in **Figure 7**. Using TreeShrink to remove possible anomalous taxa that could cause LBA before running pseudocoalescence analysis did not cause drastic differences in “before and after” topologies.

**Table 2:** Pairwise adjusted Robinson Foulds distance between each pair of tree topologies.

Tree topology	ML total	ML 75%	ASTRAL total	ASTRAL 75%	TreeShrink total	TreeShrink 75%
ML total	0	–	–	–	–	–
ML 75%	0.13	0	–	–	–	–
ASTRAL total	0.04	0.09	0	–	–	–
ASTRAL 75%	0.07	0.07	0.04	0	–	–
TreeShrink total	0.04	0.09	0	0.04	0	–
TreeShrink 75%	0.05	0.09	0.02	0.02	0.02	0

## Discussion

### Agreement Between Datasets and Analysis

The results obtained with different analyses were overall consistent, and incongruences seem to be more related to the dataset used than to the type of analysis, as indicated by RFadj values. As the level of missing data is a frequent problem in studies based on multiple markers (Huang and Lacey Knowles, 2016), we used a 75% matrix as a strategy to try to understand the effect that the high level of missing data might have on the topology and support. The effect of missing data in phylogenetic analyses has been addressed at least since fossils were included in them (Donoghue et al., 1989), and missing data are being increasingly discussed as larger datasets continue to appear. One view on the problem is that missing data do not influence the outcomes so strongly when a sufficient amount of characters has been sampled (Wiens, 2003; Wiens and Morrill, 2011).

In our analyses, reducing the number of markers decreased overall support on the trees, especially on the coalescence tree. The 75% ML analysis, besides presenting lower support, presents a major change in the position of the large South American clades, with additional changes within the clades. In the coalescence analysis, the position of the major clades remains the same, although the number of nodes with low support doubles in the 75% tree. This finding may indicate that in these two analyses, the full dataset helps to resolve internal nodes and gives more characters that support the relationships established by the cleaner dataset. However, the results of the partition analysis with PhyParts showed that removing the gene trees that are more incomplete in terms of represented taxa did improve the agreement between gene trees and species trees.

Other explanations for the differences found in the internal relationships are that some clades include a large variation in the number of loci recovered or variation in what loci were recovered in each taxon, low variation among taxa in the recovered loci, and also inadequate sampling. In Chrestinae, the most likely reason is that the recovered loci are too similar among the three species whose positions vary in different analyses, as the genus was well sampled (17 of 18 species) and the number of loci recovered for each taxon was fairly similar (271 to 307 loci with 203 being recovered for all three species). In Lychnophorinae, changes in internal relationships are likely due to the poor sampling of this diverse subtribe (only ∼18% of species sampled), and also, the fact that this subtribe seems to have diversified in a short time frame, possibly leading to low sequence divergence.

As new methods for obtaining large numbers of loci have appeared, the discussion about appropriate methods for phylogenetic inference has become a debated topic, with multiple authors advocating for the multispecies coalescence method as a more precise and biologically correct approach, as it incorporates gene tree heterogeneity that usually is ignored in analysis of concatenated matrices (Edwards et al., 2016). Overall, the phylogenetic relationships reported here are in agreement, including those recovered with different analytical methods. However, partition analysis indicates strong disagreement among gene trees and an abundance of uninformative gene trees, which improved with removal of loci that were recovered for less than 75% of the taxa present in tree.

As previously shown in a study in Cardueae, another tribe in Asteraceae, the pseudocoalescence method tends to produce trees that are more congruent in their topologies when different datasets are used (Herrando-Moraira and The Cardueae Radiations Group, 2018). We found a similar result, with the two coalescence analyses presenting only small changes in topology in internal nodes, while the reduced dataset in the concatenation analysis produced a tree with a significant change in the backbone topology. Overall support in coalescence trees seems to be largely improved by keeping a higher number of loci, even if it increases the percentage of missing data (Liu et al., 2015, Herrando-Moraira and The Cardueae Radiations Group, 2018), a result that we also observe in the current study. Removing taxa that could potentially cause LBA does not improve support in either of our trees. These results are in agreement with simulation studies, which found that pseudocoalescence methods based on gene tree topology, such as ASTRAL, are resilient to LBA effects (Roch et al., 2019).

The presence of paralogs in Asteraceae is abundant and the family has an extensively studied history of whole genome duplications (Barker et al., 2008; Barker et al., 2016; Huang et al., 2016). The probes developed by Mandel et al. (2014) used here contain a set of mostly orthologous genes; however, the phyluce pipeline still points out the recovery of possible paralogous loci in varying degrees across the species. As the probes were originally based on taxa distant from Vernonieae, we opted for completely removing any loci that could possibly present paralogy, as orthology assessment would likely be impaired by phylogenetic distance.

### Relationships Among Moquinieae, *Distephanus*, and Core Vernonieae

The present work is the first one focused on the Vernonieae that included both Moquinieae and *Distephanus*. Keeley et al. (2007) used *Distephanus* as an outgroup, while Loeuille et al. (2015a) included *Moquinia* as an outgroup. Funk and Chan (2009) investigated the influence of including different tribes and using different outgroups in the relationships within Cichorieae and usually recovered Moquinieae as the sister to the core Vernonieae, while *Distephanus* usually emerges as sister taxon to Moquinieae plus Vernonieae. Here, we present a different relationship, consistently recovered in all our trees, where *Distephanus* and Moquinieae form a clade that is sister group of all Vernonieae. Curiously, in a recently published phylogeny for the family, where part of the data presented here is also included, *Distephanus*, Moquinieae, and Vernonieae emerge sequentially in all analyses (Mandel et al., 2019). It is possible that the sampling of only one representative (*M. gigantea*) from the 165 species of Liabeae (Dillon et al., 2009) as an outgroup biased the analysis and artificially created this clade containing Moquinieae and *Distephanus*.

The two members of Moquinieae, composing two monotypic genera, have an extensive taxonomic history, due to their unusual morphology. Although they present many similarities with the Vernonieae, especially in the homogamous heads and purple florets, the inflorescence, style, and pollen morphology are starkly different from those usually found in Vernonieae and other cichorioid tribes. *M. racemosa* was firstly placed with the Gochnatieae, while *P. kingii* was initially described in Vernonieae. The two species were synonymized into *Moquinia* and placed in Vernonieae in the 1990s (Gamerro, 1990), and Robinson (1994) later placed them as separate genera in their own tribe Moquinieae. *Distephanus* also presents an unusual morphology, despite being recognized as part of the Vernonieae, with yellow flowers and trinervate leaf venation, being first placed in subtribe Liabinae (now tribe Liabeae) in the Senecioneae (Keeley and Robinson, 2009).

The phylogenetic position of this species-poor clade (tribe Moquinieae and *Distephanus*) leading to the species-rich Vernonieae potentially indicates an interesting and complicated biogeographic history, likely with multiple events of colonization of Africa and South America and extinction of lineages, as the outgroup Liabeae has an Andean distribution, while Moquinieae is exclusively Brazilian and the 50 species of *Distephanus* are distributed in Africa, India, and southern Asia. The African genera of Vernonieae have consistently been recovered as a grade leading to the New World clade (Keeley and Turner, 1990; Keeley et al., 2007), possibly indicating an initial diversification of the tribe in Africa before moving to South America again, which is in agreement with recent work (Mandel et al., 2019). Nevertheless, a detailed biogeographic study of the tribe and its closest relatives is still lacking.

### Relationships Within Vernonieae and Agreement With Past Phylogenies

Relationships within Vernonieae, especially within the South American clades, were partially contradictory in previous phylogenies (Keeley et al., 2007; Loeuille et al., 2015a) and, even after the present study, are still not completely understood. Keeley’s work (2007) has the most complete sampling in terms of genera and geographic distribution, especially regarding African and Asian genera, while the phylogeny by Loeuille et al. (2015a) expands the sampling of South American groups. Overall, the trees presented here are more similar to those found in Keeley et al. (2007).

The position of the monotypic *Stokesia* in the tribe is still a point of contention. In Keeley et al. (2007) and in the Bayesian analysis in Loeuille et al. (2015a), it is in the transition from the African to the South American Vernonieae as in our study, although in a clade with Mexican and Asian taxa. The anomalous morphology of the florets in this species, which are ligulate, and its isolated distribution in Southeastern USA might indicate that it is a leftover from a lineage that went through massive extinction, a pattern that seems frequent in Vernonieae with its abundance of monotypic genera (Keeley and Robinson, 2009).

Regarding the relationships in the South American clade, although the backbone presents wide variation among different analyses, some internal relationships remain stable. Both Keeley et al. (2007) and Loeuille et al. (2015a) recovered the same relationship between Elephantopinae and part of the Lepidaploinae. Although *Elephantopus *presents pantropical distribution, it is nested within the South American clade, with both our present work and Loeuille et al. (2015a) recovering the monotypic and strictly Brazilian *Soaresia* as its sister taxon, indicating a possible late migration from South America to other continents. Loeuille et al. (2015a) also showed the presence of Vernoniinae members, specifically *Cyrtocymura*, intermingled in Lepidaploinae, similar to the topology that we recovered here.

Keeley et al. (2007) showed a clade formed by Chrestinae and part of the Vernoniinae (*Vernonia* and *Vernonanthura*), as well as their relationship with *Heterocypsela*. In our work, we recovered a clade formed by *Heterocypsela*, *Vernonia*, and *Vernonanthura* as sister to Chrestinae, while in Keeley et al. (2007), *Heterocypsela* emerges as most closely related to *Chresta*. This previous work included two genera not sampled here, *Tephrothamnus* and *Eirmocephala*, from South and Central America, which could change the relationships we found if included. The relationship of *Chresta* with other Vernonieae has always been unclear (Robinson, 1992), as the genus presents secondary heads, which approximate it to the Lychnophorinae but also pollen and anther appendage features (Robinson, 1999a) that suggest a closer relationship to Vernoniinae.

Loeuille et al. (2015a) postulated the multiple origins of syncephaly in the Vernonieae, deeming classifications based on this character artificial. In the trees presented here, *Chresta* indeed is closer to other taxa lacking secondary heads than to Lychnophorinae, indicating the complex evolution of secondary heads, possibly through different developmental steps.

The relationship of the large clade formed by CHR + VER with the other Vernonieae varies depending on the analysis and dataset, although most trees agree with CHR + VER being the sister group of ELE + LEP + VER (**Figure 4**), although with low support. The exception is the ML analysis with the 75% dataset (**Figure 4A**), which shows CHR + VER as the sister group of Lychnophorinae. In all other analyses, LYC emerges as the sister group of (CHR + VER) + (ELE + LEP + VER). Keeley’s phylogeny (2007) agrees with our 75% ML analysis, with (CHR + VER) + LYC and ELE + LEP + VER as the sister clade of this larger clade.

In Loeuille’s work (2015a), Chrestinae and *Stokesia* emerge as sister to a clade formed by LYC and *Vernonia + Vernonanthura*. The bulk of LEP groups with ELE and some other VER, forming the sister clade of CHR + (*Vernonia + Vernonanthura* + LYC). None of the trees in the present work support these relationships.

When subtribe Chrestinae was created (Robinson, 1992; Robinson, 1999a), the monotypic genus *Soaresia* from Central Brazil was placed in it due to some morphological similarities, mainly the presence of secondary head and pollen type. However, in Loeuille’s work (2015a), *Soaresia* emerges as the sister taxon of the Elephantopinae, with the same relation shown in all analyses presented here. In fact, *Soaresia* has morphological affinities to *Elephantopus*, such as the bristle-like awls that compose the pappus and the unbranched trichomes, further supporting its transference to subtribe Elephantopinae (Loeuille et al., 2015a).

Also, the analyses presented here do not support the monophyly of subtribe Dipterocypselinae. This subtribe was created to accommodate two monotypic genera that present dimorphic cypsela (*Dipterocypsela* and *Heterocypsela*) and a third monotypic genus (*Manyonia*) without dimorphic cypsela (Keeley and Robinson, 2009), with a fourth monotypic genus (*Allocephalus*) with dimorphic cypsela being added later (Bringel Jr et al., 2011). We sampled only the two Brazilian representatives of the subtribe, *Heterocypsela* and *Allocephalus*, both from Central Brazil and growing on limestone outcrops. *Dipterocypsela* is found on Northern Colombia, also on limestone outcrops (Blake, 1945). *Manyonia* does not present fruit dimorphism, but the inflorescence structure and the pattern of the cells on the cypsela walls placed it close to *Heterocypsela* and *Dipterocypsela* (Robinson, 1999b), regardless of this species being known only in Tanzania. *Heterocypsela* and *Allocephalus* fall in distant places in our trees, in the Vernoniinae and Lychnophorinae, respectively. Due to the morphological singularities of these four genera, their placement within Vernonieae subtribes has always been putative at best (Blake, 1945, Robinson, 1999b), and its status as a subtribe should be reevaluated, depending on the inclusion of *Dipterocypsela* and *Manyonia* in future analyses.

Another finding from our analyses is the non-monophyly of both Vernoniinae and Lepidaploinae. As sampled here, Lepidaploinae terminals emerged in two clades, one including *Cyrtocymura*, which is currently placed in Vernoniinae, and another sister to Elephantopinae. Vernoniinae terminals also emerged separated, with *Vernonia* and *Vernonanthura* being sister to *Chresta*, and *Cyrtocymura* grouping with the LEP + ELE. These separations had already been shown in Loeuille’s analysis (2015a), although with lower resolution and support. Lepidaploinae was initially included as a complex of genera within Vernoniinae (Robinson, 1999a), later being separated due to complex combinations of micro- and macrocharacters (Keeley and Robinson, 2009), such as the echinolophate pollen and the seriate-cymose inflorescences. Although combinations of characters can be useful for identification of genera and species, it is becoming clear that many of them are homoplastic, producing classifications that do not reflect the evolutionary history, and this seems to be the case in the infra-tribal classification in Vernonieae, which will have to be reevaluated as more inclusive analyses become available.

Regarding Lychnophorinae, the relations uncovered here slightly differ from those seen in Loeuille et al. (2015b); however, these differences are difficult to interpret due to our low taxonomic sampling, which includes only a few representatives from each major clade within it. As previously shown by Loeuille et al. (2015a, 2015b), Centratherinae emerges as the sister taxon of all other Lychnophorinae and is now considered a synonym (Loeuille et al., 2019), as well as Sipolisiinae, whose members emerge in several positions within Lychnophorinae. The monotypic *Allocephalus*, not included in previous phylogenies, here emerges as sister to the rest of Lychnophorinae. It displays various plesiomorphic features of Lychnophorinae: herbaceous habit (*Centratherum*), T-shaped trichomes (*Albertinia*, *Centratherum*, etc.), and heads in dense glomerules (*Blanchetia*, *Gorceixia*). It shares with *Albertinia* a style with basal node (feature uncommon in Lychnophorinae) and especially, as noted by Bringel Jr et al. (2011), an involucre with fused phyllaries.

This peculiar involucre sheds an interesting light on the origin of the unique alveolate receptacle of *Albertinia* that has been variously interpreted: Candolle (1836) assumed that *Albertinia* had one floret per capitulum and fused capitula as in *Eremanthus* and *Lychnophora*, but since Schultz-Bipontinus (1861, 1863), *Albertinia* capitula are interpreted as multiflowered and the receptacle surface with deep holes (alveolae) (Robinson, 1999a, Loeuille et al., 2015a). More studies are clearly necessary, but the position of *Allocephalus* as sister group of Lychnophorinae calls to reevaluate the morphological interpretation of the “capitulum” of *Albertinia* and indicates further directions to study the evolution in syncephaly in Lychnophorinae.

The clade grouping *Chronopappus*, *Heterocoma*, *Maschalostachys*, and *Paralychnophora* was also recovered by Loeuille et al. (2015b) but only in one analysis (Bayesian analysis without morphological data). However, it appeared as the sister group of the *Prestelia* Alliance clade (*E. crotonoides* + *Hololepis*) in that study, instead of sister to the derived Lychnophorinae genera, as seen in the present analysis. Similarly to previous phylogenies (Loeuille et al., 2015a, Loeuille et al., 2015b), *Minasia*, *Lychnophorella*, *Piptolepis*, *Lychnophora*, and *Eremanthus* are grouped in a large clade, but its internal relationships vary between the analyses.

Our work did not sample Piptocarphinae, a mainly South American subtribe that includes more than 50 species. Loeuille’s work (2015a) shows that the subtribe has affinities with Vernoniinae, Lepidaploinae, and Elephantopinae, although without resolution, indicating this might be a crucial group to help resolving the relationships in the South American clade. Also, as shown by Keeley et al. (2007), the relationships in the African clade are complex, especially close to the transition to South America and should be further investigated with additional sampling, which might help to solve the position of *Stokesia* in relation to the Old and New World clades.

## Conclusions

The Hyb-Seq method used to obtain sequence data for phylogenetic reconstruction proved useful and powerful, allowing us to recover well-resolved and supported relationships in Vernonieae. We consistently recovered the same overall topology regardless of dataset and analysis method, even with incongruence among gene and species trees, with most of the effect of reducing the dataset being the overall decline in statistical support in the tree. Also, we demonstrated the non-monophyly of several subtribes, indicating that further phylogenetic and taxonomic work should be conducted, and that the circumscription of tribe Moquinieae and genus *Distephanus* should be probably reevaluated in relation to their affinity with Vernonieae. The presence of more than 50 monotypic genera in Vernonieae (Keeley and Robinson, 2009) complicates phylogenetic studies, making the sampling process very challenging and possibly indicating an evolutionary history of multiple speciation and extinction events. On the other hand, more complete sampling in future studies may reveal strongly supported clades that could eventually allow a reduction of the number of monotypic genera recognized in the tribe. While the recently developed Hyb-Seq method proved to be reliable, further investigation into Vernonieae phylogeny should focus in improving sampling, especially in lineages that are isolated or morphologically anomalous.

## Data Availability Statement

Raw data in the fastq format are deposited at the NCBI Sequence Read Archive, under BioProjects PRJNA540287 and PRJNA546287.

## Author Contributions

CS designed the study, collected samples, conducted lab work, conducted data analyses, and wrote the manuscript. BL helped designed the study, participated in field collections, provided samples, helped write the discussion section, and reviewed and commented on the manuscript. VF provided samples and reviewed and commented on the manuscript. JM provided samples, collaborated on lab work, data analyses, and reviewed and commented on the manuscript. JP helped design the study and commented and reviewed the manuscript, besides being the thesis advisor for CS’s doctoral dissertation.

## Funding

This work was funded by the Fundação de Amparo a Pesquisa do Estado da São Paulo doctoral scholarships 2013/18189-2 and 2016/12446-1 and by the National Science Foundation Division of Environmental Biology, grant DEB-1745197.

## Conflict of Interest

The authors declare that the research was conducted in the absence of any commercial or financial relationships that could be construed as a potential conflict of interest.

## Supplementary Material

Supplementary Table 1 Voucher list of taxa sampled in the study, containing collection data and first date of publication of each sequence.

Supplementary Table 2 General information about the matrices used for phylogenetic analyses, number and identity of loci recovered and molecular evolution models used in gene tree assembly.

## References

[B1] Bankevich A. Nurk S. Antipov D. Gurevich A. Dvorkin M. Kulikov A. S. (2012). SPAdes, A new genome assembly algorithm and its applications to single-cell sequencing. J. Comput. Biol. 19 (5), 455–477. 10.1089/cmb.2012.0021 22506599 PMC3342519

[B2] Barker M. S. Kane N. C. Matvienko M. Kozik A. Michelmore R. W. Knapp S. J. (2008). Multiple paleopolyploidizations during the evolution of the Asteraceae reveal parallel patterns of duplicate gene retention after millions of years. Mol. Biol. Evol. 25 (11), 2445–2455. 10.1093/molbev/msn187 18728074 PMC2727391

[B3] Barker M. S. Li Z. Kidder T. I. Reardon C. R. Lai Z. Oliveira L. O. (2016). Most Compositae (Asteraceae) are descendants of a paleohexaploid and all share a paleotetraploid ancestor with the Calyceraceae. Am. J. Bot. 103 (7), 1203–1211. 10.3732/ajb.1600113 27313199

[B4] Blake S. F. (1945). *Dipterocypsela*, a new genus of Vernonieae from Colombia. J. Wash. Acad. Sci. 35 (2), 36–38.

[B5] Bolger A. M. Lohse M. Usadel B. (2014). Trimmomatic, A flexible trimmer for Illumina Sequence Data. Bioinformatics 30 (15), 2114–2120. 10.1093/bioinformatics/btu170 24695404 PMC4103590

[B6] Borowiec M. L. (2016). AMAS: a fast tool for alignment manipulation and computing of summary statistics. PeerJ 4, e1660. 10.7717/peerj.1660 26835189 PMC4734057

[B7] Bremer K. (1994). Asteraceae: Cladistics & Classification. Portland: Timber Press.

[B8] Bringel J. B. A. Jr. Nakajima J. N. Robinson H. (2011). *Allocephalus gamolepis*, a new genus and species of Dipterocypselinae (Vernonieae, Asteraceae) from Central Brazil. Syst. Bot. 36 (3), 785–788. 10.1600/036364411X583736

[B9] Brown J. W. Walker J. F. Smith S. A. (2017). Phyx: phylogenetic tools for unix. Bioinformatics 33 (12), 1886–1888. 10.1093/bioinformatics/btx063 28174903 PMC5870855

[B10] Candolle A. P. (1836). “Vernoniaceae,” in Prodromus Systematis Naturalis Regni Vegetabilis, vol. v. 5 . Ed. de Candolle A. P. (Paris: Masson, Paris: Treutel and Würtz), 9–103.

[B11] Cassini H. (1819). Suite du sixième mémoire sur la famille des Synanthérées, contenant les caractères des tribus. J. Phys. Chim. Hist. Nat. Arts 88, 189–204.

[B12] Cronn R. Knaus B. J. Liston A. Maughan P. J. Parks M. Syring J. V. (2012). Targeted enrichment strategies for next-generation plant biology. Am. J. Bot. 99 (2), 291–311. 10.3732/ajb.1100356 22312117

[B13] Darriba D. Taboada G. L. Doallo R. Posada D. (2012). jModelTest 2, more models, new heuristics and parallel computing. Nat. Methods 9 (8), 772. 10.1038/nmeth.2109 PMC4594756 22847109

[B14] Dillon M. O. Funk V. A. Robinson H. Chan R. (2009). “Liabeae,” in Systematics, Evolution, and Biogeography of Compositae. Eds. Funk V. A. Susanna A. Stussey T. F. Bayer R. J. (Vienna, Austria: International Association for Plant Taxonomy (IAPT)), 417–437.

[B15] Donoghue M. J. Doyle J. A. Gauthier J. Kluge A. G. Rowe T. (1989). The importance of fossils in phylogeny reconstruction. Annu. Rev. Ecol. Evol. Syst. 20, 431–460. 10.1146/annurev.es.20.110189.002243

[B16] Edwards S. V. Xi Z. Jnake A. Faircloth B. C. McCormack J. E. Glenn T. C. (2016). Implementing and testing the multispecies coalescent module: a valuable paradigm for phylogenomics. Mol. Phylogenet. Evol. 94, 447–462. 10.1016/j.ympev.2015.10.027 26518740

[B17] Faircloth B. C. McCormack J. E. Crawford N. G. Harvey M. G. Brumfield R. T. Glenn T. C. (2012). Ultraconserved elements anchor thousands of genetic markers spanning multiple evolutionary timescales. Syst. Biol. 61 (5), 717–726. 10.1093/sysbio/sys004 22232343

[B18] Faircloth B. C. (2016). PHYLUCE is a software package for the analysis of conserved genomic loci. Bioinformatics 32, 786–788. 10.1093/bioinformatics/btv646 26530724

[B19] Folk R. A. Mandel J. R. Freudenstein J. V. (2015). A protocol for targeted enrichment of intron-containing sequence markers for recent radiations: A phylogenomic example from *Heuchera* (Saxifragaceae). Appl. Plant Sci. 3 (8), 1500039. 10.3732/apps.1500039 PMC4542943 26312196

[B20] Funk V. A. Anderberg A. A. Baldwin B. G. Bayer R. J. Bonifacino J. M. Breitwieser I. (2009). “Compositae metatrees: The next generation,” in Systematics, Evolution, and Biogeography of Compositae. Eds. Funk V. A. Susanna A. Stussey T. F. Bayer R. J. (Vienna, Austria: International Association for Plant Taxonomy (IAPT)), 747–777.

[B21] Funk V. A. Chan R. (2009). “Introduction to Cichorioideae,” in Systematics, Evolution, and Biogeography of Compositae. Eds. Funk V. A. Susanna A. Stussey T. F. Bayer R. J. (Vienna, Austria: International Association for Plant Taxonomy (IAPT)), 335–342.

[B22] Gamerro J. C. (1990). Identidad de *Pseudostifftia* con *Moquinia* (Compositae) y consideraciones sobre la ubicación tribal del taxon. Darwiniana 30, 123–136.

[B23] Grover C. E. Salmon A. Wendel J. F. (2012). Targeted sequence capture as a powerful tool for evolutionary analysis. Am. J. Bot. 99 (2), 312–319. 10.3732/ajb.1100323 22268225

[B24] Guindon S. Gascuel O. (2003). A simple, fast and accurate method to estimate large phylogenies by maximum-likelihood. Syst. Biol. 52, 696–704. 10.1080/10635150390235520 14530136

[B25] Herrando-Moraira S. The Cardueae Radiations Group (2018). Exploring data processing strategies in NGS target enrichment to disentangle radiations in the tribe Cardueae (Compositae). Mol. Phylogenet. Evol. 128, 69–87. 10.1016/j.ympev.2018.07.012 30036700

[B26] Huang C. H. Zhang C. Liu M. Gao T. Qi J. Ma H. (2016). Multiple polyploidization events across Asteraceae with two nested events in the early history revealed by nuclear phylogenomics. Mol. Biol. Evol. 33 (11), 2820–2835. 10.1093/molbev/msw157 27604225 PMC5062320

[B27] Huang H. Lacey Knowles L. (2016). Unforeseen consequences of excluding missing data from next-generation sequences, simulation studies of RAD sequences. Syst. Biol. 65 (3), 357–365. 10.1093/sysbio/syu046 24996413

[B28] Jansen R. K. Palmer J. D. (1988). Phylogenetic implications of chloroplast DNA restriction site variation in the Mutisieae (Asteraceae). Am. J Bot. 75, 751–764. 10.1002/j.1537-2197.1988.tb13496.x 30139093

[B29] Jeffrey C. (1988). The Vernonieae of east tropical Africa. Notes on the Compositae: V. Kew Bull. 43, 195–277. 10.2307/4113734

[B30] Jones S. B. (1979). Synopsis and pollen morphology of *Vernonia* (Compositae, Vernonieae) in the New World. Rhodora 8, 425–447.

[B31] Jones S. B. (1981). Synoptic classification and pollen morphology of *Vernonia* (Compositae: Vernonieae) in the Old World. Rhodora 83, 59–75.

[B32] Johnson M. (2017). https://github.com/mossmatters/phyloscripts/tree/master/phypartspiecharts.

[B33] Johnson M. Pokorny L. Dodsworth S. Botigue L. R. Cowan R. S. Devault A. (2019). A Universal Probe Set for Targeted Sequencing of 353 Nuclear Genes from Any Flowering Plant Designed Using k-medoids Clustering. Syst. Biol. 68, 695–699. 10.1093/sysbio/syy086 PMC6568016 30535394

[B34] Karis P. O. Funk V. A. McKenzie R. J. Barker N. P. Chan R. (2009). “Arctotideae,” in Systematics, Evolution, and Biogeography of Compositae. Eds. Funk V. A. Susanna A. Stussey T. F. Bayer R. J. (Vienna, Austria: International Association for Plant Taxonomy (IAPT)), 385–410.

[B35] Keeley S. C. Turner B. L. (1990). A preliminary cladistic analysis of the genus *Vernonia* (Vernonieae: Asteraceae). Plant Syst. Evol. 4, 45–66. 10.1007/978-3-7091-6928-5_3

[B36] Keeley S. C. Forsman Z. H. Chan R. (2007). A phylogeny of the “evil tribe” (Vernonieae, Compositae) reveals Old/New World long distance dispersal: support from separate and combined congruent datasets (trnL-F, ndhF, ITS). Mol. Phylogenet. Evol. 44, 89–103. 10.1016/j.ympev.2006.12.024 17292633

[B37] Keeley S. C. Robinson H. (2009). “Vernonieae,” in Systematics, Evolution, and Biogeography of Compositae. Eds. Funk V. A. Susanna A. Stussey T. F. Bayer R. J. (Vienna, Austria: International Association for Plant Taxonomy (IAPT)), 439–469.

[B38] Lanfear R. Calcott B. Ho S. Y. W. Guindon S. (2012). PartitionFinder, combined selection of partitioning schemes and substitution models for phylogenetic analyses. Mol. Biol. Evol. 29 (6), 1695–1701. 10.1093/molbev/mss020 22319168

[B39] Lanfear R. Calcott B. Kainer D. Mayer C. Stamatakis A. (2014). Selecting optimal partitioning schemes for phylogenomic datasets. BMC Evol. Biol. 14 (1), 82. 10.1186/1471-2148-14-82 24742000 PMC4012149

[B40] Liu L. Xi Z. Wu S. Davis C. C. Edwards S. V. (2015). Estimating phylogenetic trees from genome-scale data. Ann. N. Y. Acad. Sci. 1360, 36–53. 10.1111/nyas.12747 25873435

[B41] Loeuille B. Keeley S. C. Pirani J. R. (2015a). Systematics and evolution of syncephaly in American Vernonieae (Asteraceae) with emphasis on the Brazilian subtribe Lychnophorinae. Syst. Bot. 40 (1), 286–298. 10.1600/036364415X686576

[B42] Loeuille B. Semir J. Lohmann L. G. Pirani J. R. (2015b). A phylogenetic analysis of Lychnophorinae (Asteraceae, Vernonieae) based on molecular and morphological data. Syst. Bot. 40 (1), 299–315. 10.1600/036364415X686585

[B43] Loeuille B. Semir J. Pirani J. R. (2019). A synopsis of Lychnophorinae (Asteraceae: Vernonieae). Phytotaxa 398, 1–139. 10.11646/phytotaxa.398.1.1

[B44] Mai U. Mirarab S. (2018). TreeShrink: fast and accurate detection of outlier long branches in collections of phylogenetic trees. BMC Genomics 19 (S5), 272. 10.1186/s12864-018-4620-2 29745847 PMC5998883

[B45] Mandel J. R. Dikow R. B. Funk V. A. Masalia R. R. Evan Staton S. Kozik A. (2014). A target enrichment method for gathering phylogenetic information from hundreds of loci, an example from the Compositae. Appl. Plant Sci. 2 (2), 1300085. 10.3732/apps.1300085 PMC4103609 25202605

[B46] Mandel J. R. Barker M. S. Bayer R. J. Dikow R. B. Gao T. G. Jones K. E. (2017). The Compositae tree of life in the age of phylogenomics. J. Syst. Evol. 55 (4), 405–410. 10.1111/jse.12265

[B47] Mandel J. R. Dikow R. B. Siniscalchi C. M. Thapa R. Watson L. E. Funk V. A. (2019). A fully resolved backbone phylogeny reveals numerous dispersals and explosive diversifications throughout the history of Asteraceae. Proc. Natl. Acad. Sci. 116 (28), 14083–14088. 10.1073/pnas.1903871116 31209018 PMC6628808

[B48] Mitchell N. Lewis P. O. Lemmon E. M. Lemmon A. R. Holsinger K. E. (2017). Anchored phylogenomics improves the resolution of evolutionary relationships in the rapid radiation of *Protea* L. Am. J. Bot. 104, 102–115. 10.3732/ajb.1600227 28104589

[B49] Nicholls J. A. Pennington R. T. Koenen E. J. M. Hughes C. E. Hearn J. Bunnefeld L. (2015). Using targeted enrichment of nuclear genes to increase phylogenetic resolution in the neotropical rain forest genus *Inga* (Leguminosae: Mimosoideae). Front. Plant Sci. 6, 710. 10.3389/fpls.2015.00710 26442024 PMC4584976

[B50] Panero J. L. Funk V. A. (2008). The value of sampling anomalous taxa in phylogenetic studies: major clades of the Asteraceae revealed. Mol. Phylogenet. Evol. 47, 757–782. 10.1016/j.ympev.2008.02.011 18375151

[B51] Robinson H. (1992). Notes on the Lychnophorinae from Minas Gerais, Brazil, a synopsis of *Lychnophoriopsis* Schultz-Bip., and the new genera *Anteremanthus* and *Minasia* (Vernonieae, Asteraceae). Proc. Biol. Soc. Wash. 105, 640–652.

[B52] Robinson H. (1994). Notes on the tribes Eremothamneae, Gundelieae and Moquinieae, with comparisons of their pollen. Taxon 43, 33–44. 10.2307/1223458

[B53] Robinson H. (1999a). Generic and subtribal classification of American Vernonieae. Smithsonian Contr. Bot. 89, 1–116. 10.5479/si.0081024X.89

[B54] Robinson H. (1999b). Revisions in paleotropical Vernonieae (Asteraceae). Proc. Biol. Soc. Wash. 112, 220–247.

[B55] Roch S. Nute M. Warnow T. (2019). Long-branch attraction in species tree estimation: inconsistency of partitioned likelihood and topology-based summary methods. Syst. Biol. 68, 281–297. 10.1093/sysbio/syy061 30247732

[B56] Schultz-Bipontinus C. H. (1861). Cassiniaceae uniflorae, oder Verzeichniss der Cassiniaceen mit 1-blüthigen Köpfchen. Jahresbericht der Pollichia 18/19, 157–190.

[B57] Schultz-Bipontinus C. H. (1863). *Lychnophora* Martius! und einige benachbarte Gattungen. Jahresbericht der Pollichia 20/21, 321–439.

[B58] Semple J. C. Watanabe K. (2009). “A review of chromosome numbers in Asteraceae with hypotheses on chromosomal base number evolution,” in Systematics, Evolution, and Biogeography of Compositae. Eds. Funk V. A. Susanna A. Stussey T. F. Bayer R. J. (Vienna, Austria: International Association for Plant Taxonomy (IAPT)), 61–72.

[B59] Smith S. A. Moore M. J. Brown J. W. Yang Y. (2015). Analysis of phylogenomic datasets reveals conflict, concordance, and gene duplications with examples from animals and plants. BMC Evol. Biol. 15, 150. 10.1186/s12862-015-0423-0 26239519 PMC4524127

[B60] Stamatakis A. (2006). RAxML-VI-HPC, maximum likelihood-based phylogenetic analyses with thousands of taxa and mixed models. Bioinformatics 22, 2688–2690. 10.1093/bioinformatics/btl446 16928733

[B61] Stamatakis A. (2014). RAxML Version 8, A tool for Phylogenetic Analysis and Post-Analysis of Large Phylogenies. Bioinformatics 30 (9), 1312–1313. 10.1093/bioinformatics/btu033 24451623 PMC3998144

[B62] Swofford D. L. (2003). PAUP*: phylogenetic analysis using parsimony, version 4.0 b10.

[B63] de Tournefort J. P. (1700). Institutiones Rei Herbariae Vol. 3 vols. Paris: Typographia Regia. 10.5962/bhl.title.713

[B64] Vaillant S. (1719-1723). Établissement de nouveaux caractères de trois familles ou classes de plantes à fleurs composées; sçavoir, des Cynarocéphales, des Corymbifères, et des Cichoracées. Mémoires de l’Académie Royale des Sciences.

[B65] Wen J. Egan A. N. Dikow R. B. Zimmer E. A. (2015). “Utility of transcriptome sequencing for phylogenetic inference and character evolution,” in Next Generation Sequencing in Plant Systematics. Eds. Hörandl E. Apperlhans M. S. (Vienna, Austria: International Association for Plant Taxonomy (IAPT)).

[B66] Wiens J. J. (2003). Missing data, incomplete taxa, and phylogenetic accuracy. Syst. Biol. 52 (4), 528–538. 10.1080/10635150390218330 12857643

[B67] Wiens J. J. Morrill M. C. (2011). Missing data in phylogenetic analysis: reconciling results from simulations and empirical data. Syst. Biol. 60 (5), 719–731. 10.1093/sysbio/syr025 21447483

[B68] Zhang C. Rabiee M. Sayyari E. Mirarab S. (2018). ASTRAL-III: polynomial time species tree reconstruction from partially resolved gene trees. BMC Bioinformatics 19 (6), 153. 10.1186/s12859-018-2129-y 29745866 PMC5998893

