# Supplementary material for: Phylogenomics Yields New Insight Into Relationships Within Vernonieae (Asteraceae)
Source: Front Plant Sci. 2019 Oct 17;10:1224. doi: 10.3389/fpls.2019.01224 (PMC6843069; doi:10.3389/fpls.2019.01224)
Supplement: Supplementary file 1 [file Image_1.pdf]

Supplementary Material

1.1 Supplementary Figures

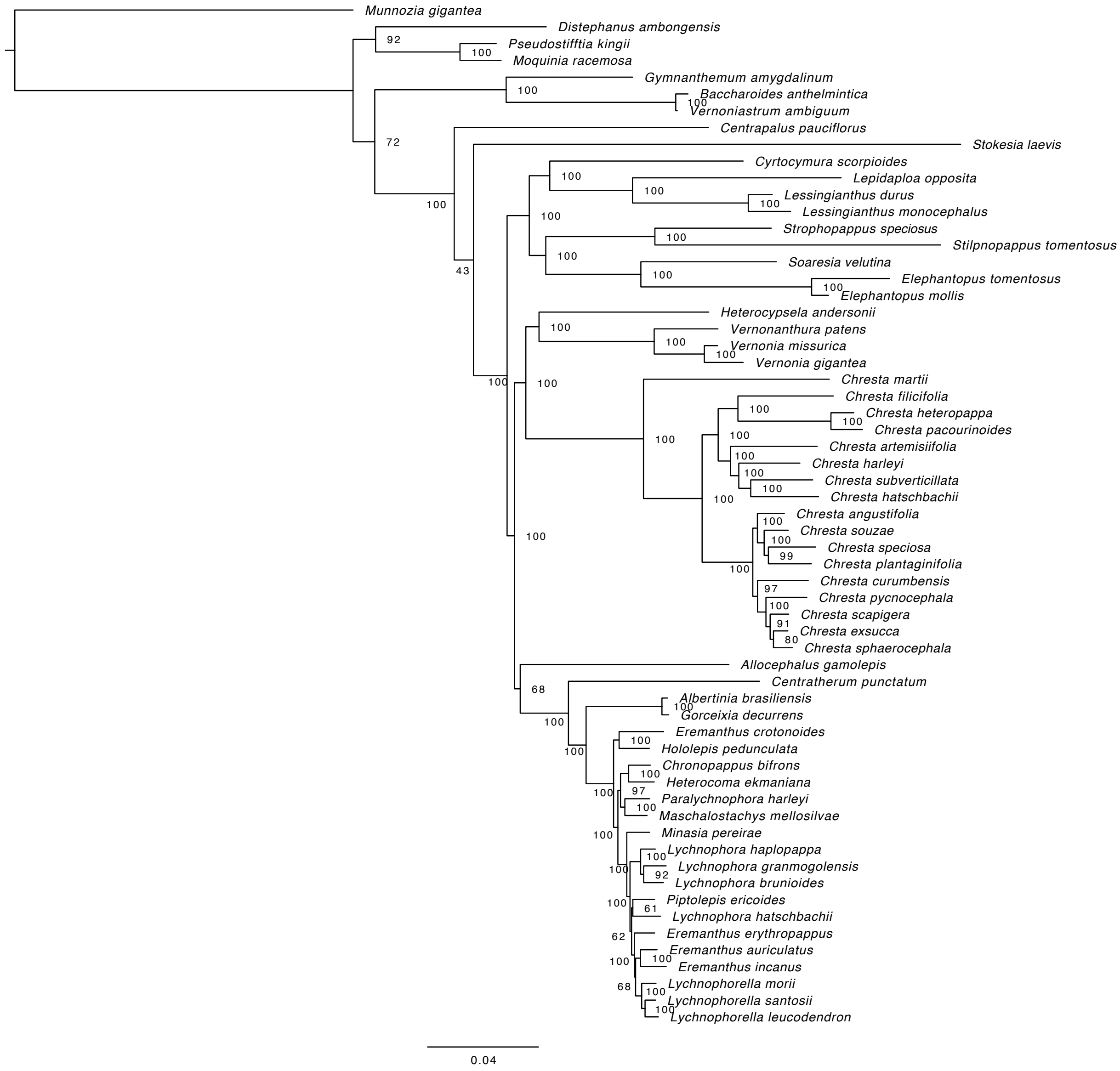

Supplementary Figure 1. Maximum likelihood tree obtained with the 75% matrix (113,347 characters), with model GTR+G+I, with 1000 bootstrap replicates. Node values are bootstrap values.

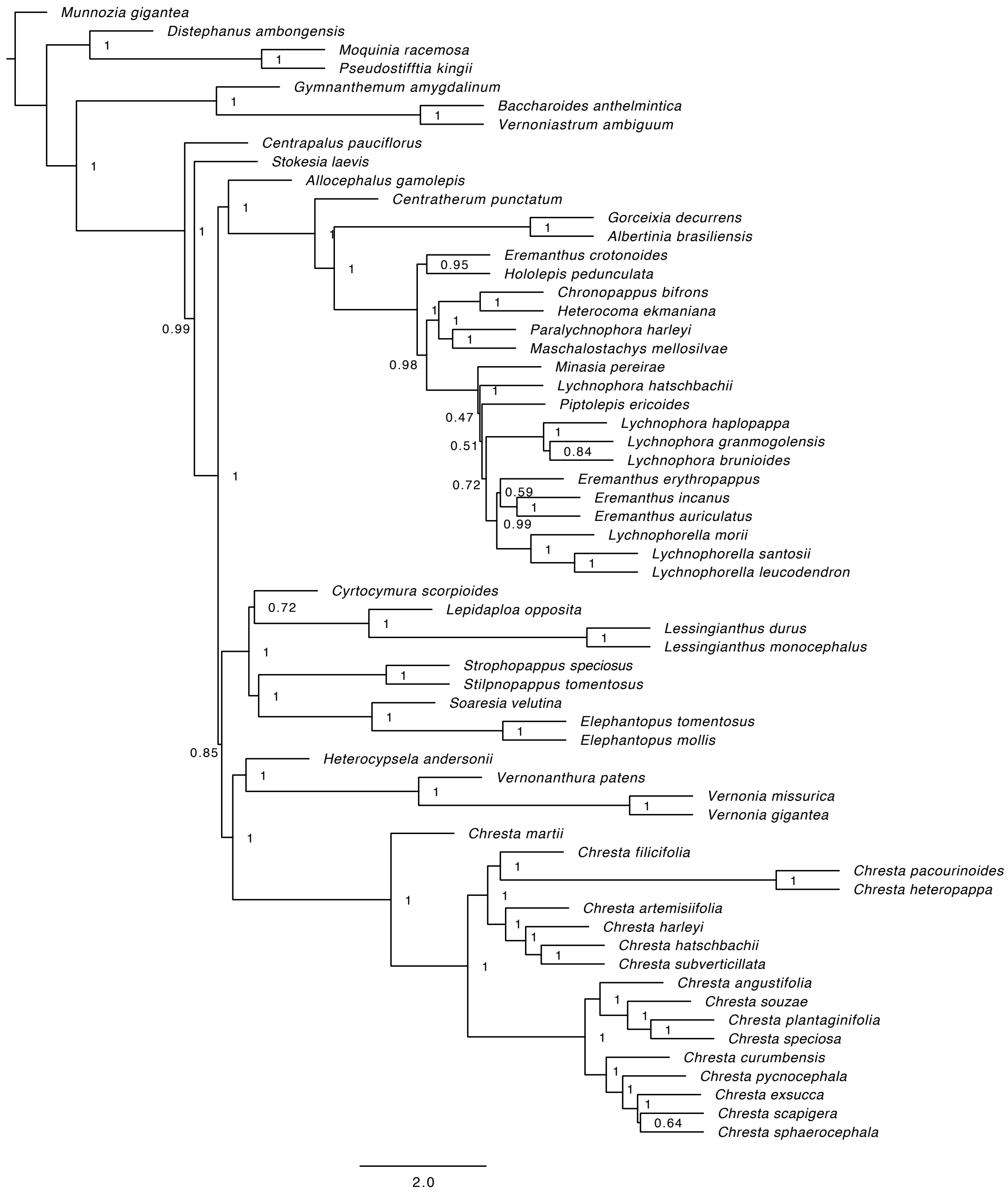

Supplementary Figure 2. Pseudocoalescence tree obtained with the total matrix (645 loci). Node values are local posterior probability.

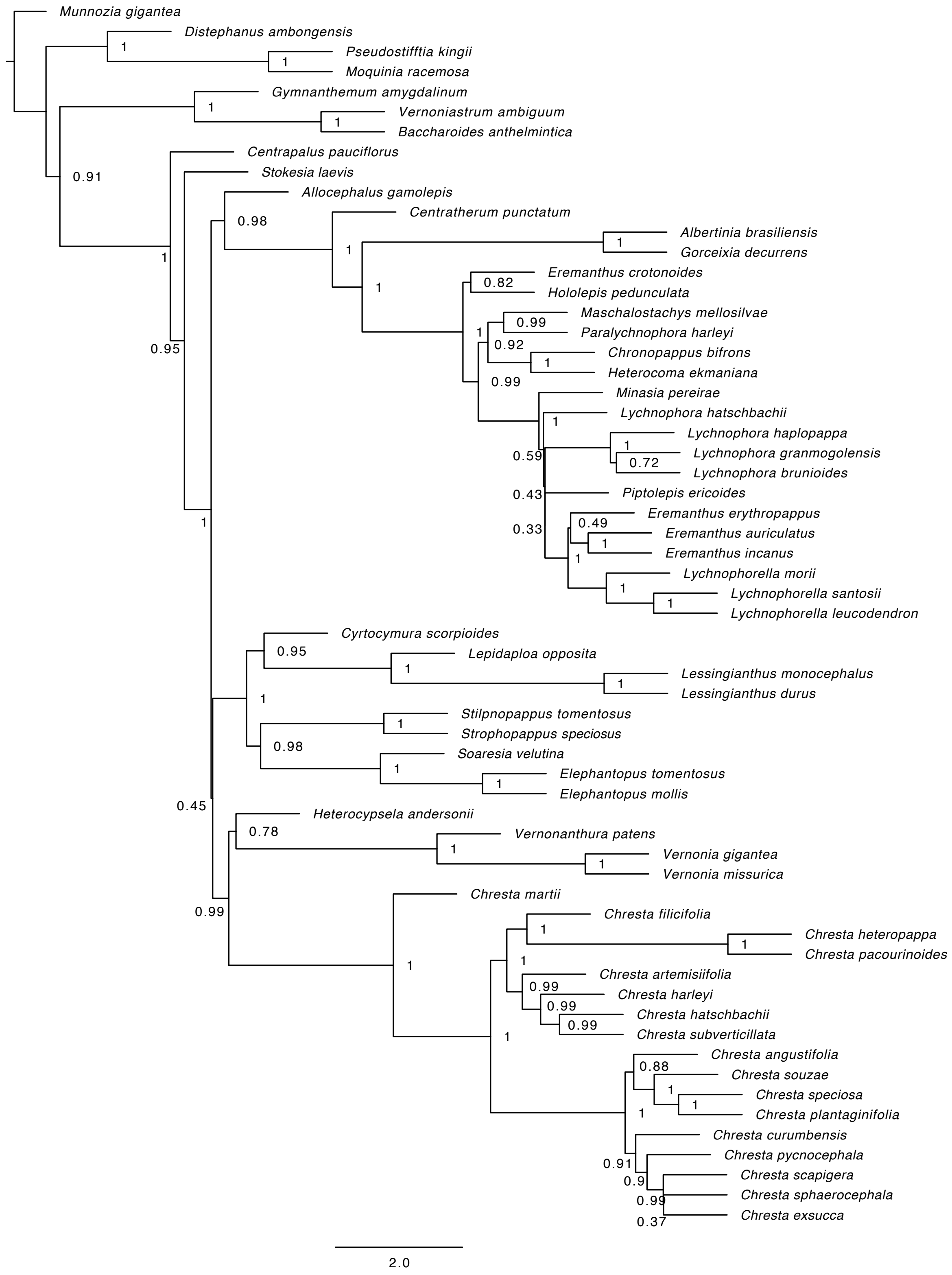

Supplementary Figure 3. Pseudocoalescence tree obtained with the 75% matrix (87 loci). Node values are local posterior probability.

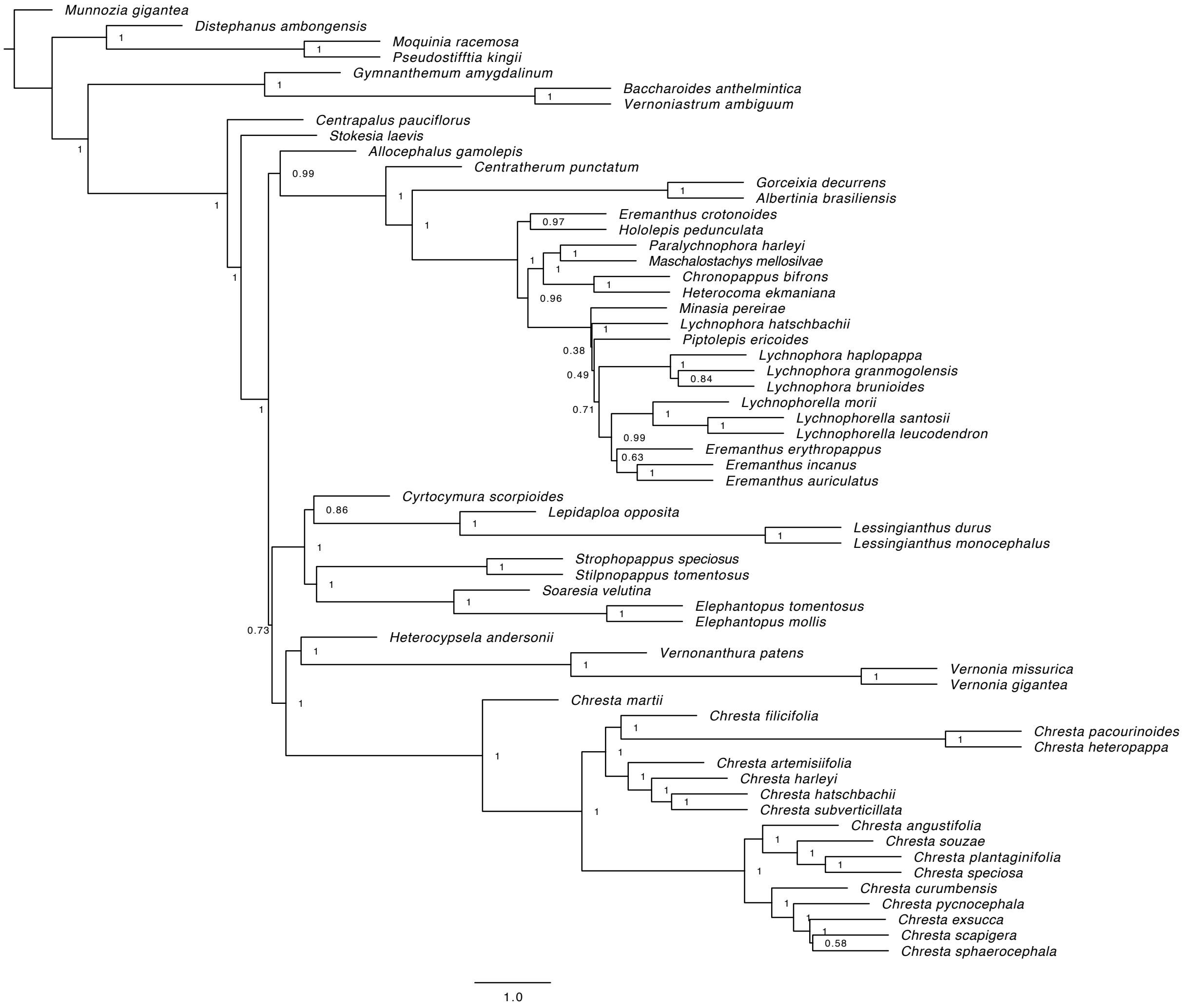

Supplementary Figure 4. Pseudocoalescence tree obtained with the total matrix after treatment of the genes tree with TreeShrink and removing taxa that could cause long-branch attraction. Node values are local posterior probability.

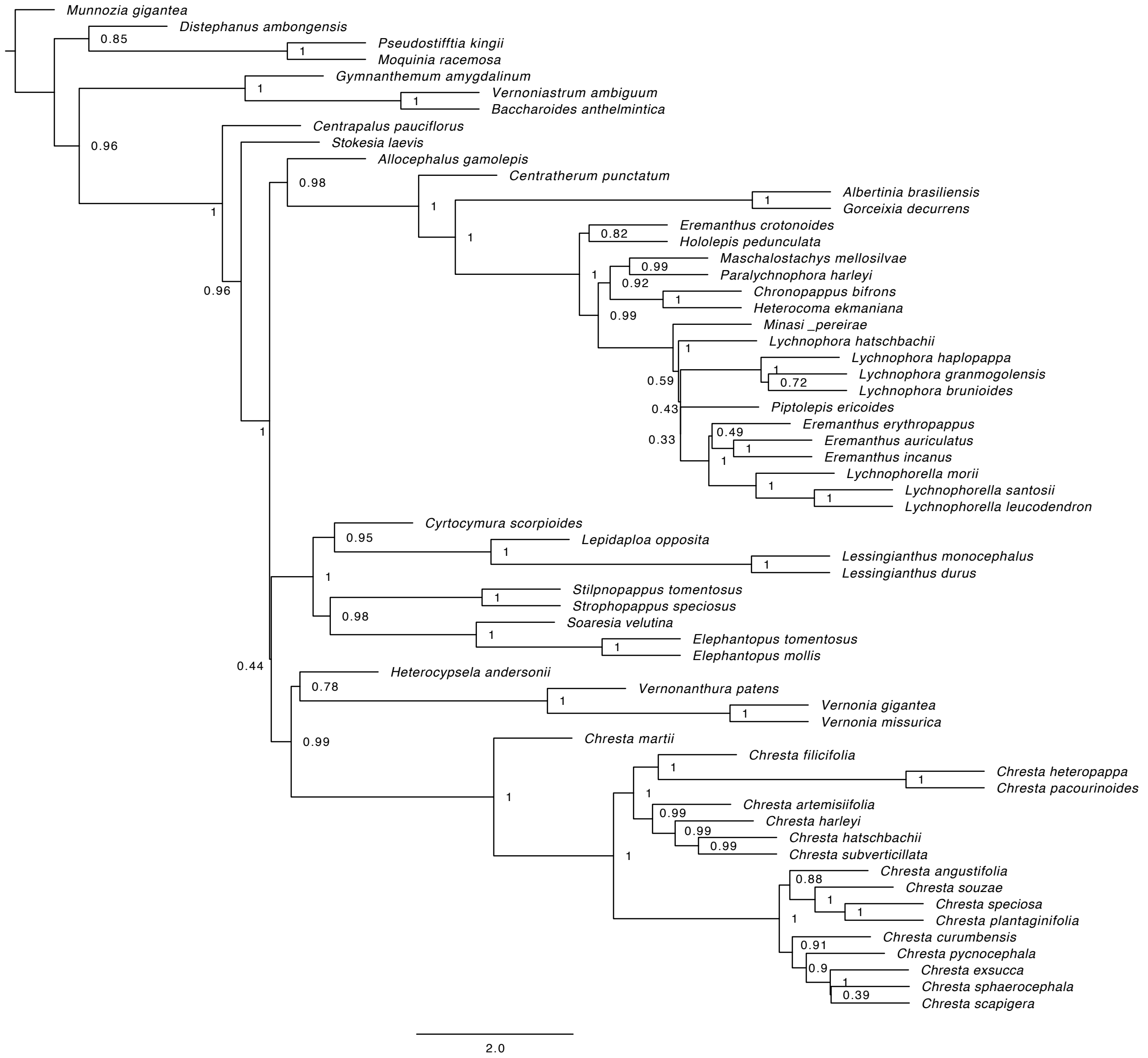

Supplementary Figure 5. Pseudocoalescence tree obtained with the 75% matrix after treatment of the genes tree with TreeShrink and removing taxa that could cause long-branch attraction. Node values are local posterior probability.
