# Supplementary material for: Phylogenomics Yields New Insight Into Relationships Within Vernonieae (Asteraceae)
Source: Front Plant Sci. 2019 Oct 17;10:1224. doi: 10.3389/fpls.2019.01224 (PMC6843069; doi:10.3389/fpls.2019.01224)
Supplement: Supplementary file 4 [file Table_3.docx]

Supplementary Material

## Supplementary Tables

**Table 3.** Number of trees from which each taxon was removed due to possible long-branch attraction effects as defined by TreeShrink (α = 0.05).

|  | No. trees where taxon was removed (total) | No. trees where taxon was removed (75%) |
| --- | --- | --- |
| *Albertinia brasiliensis* | 3 | 0 |
| *Allocephalus gamolepis* | 15 | 1 |
| *Baccharoides anthelmintica* | 21 | 1 |
| *Centrapalus pauciflorus* | 24 | 0 |
| *Centratherum punctatum* | 10 | 0 |
| *Chresta angustifolia* | 11 | 0 |
| *Chresta artemisiifolia* | 13 | 0 |
| *Chresta curumbensis* | 16 | 0 |
| *Chresta exsucca* | 3 | 0 |
| *Chresta filicifolia* | 15 | 0 |
| *Chresta harleyi* | 4 | 0 |
| *Chresta hatschbachii* | 10 | 0 |
| *Chresta heteropappa* | 15 | 0 |
| *Chresta martii* | 12 | 0 |
| *Chresta pacourinoides* | 14 | 1 |
| *Chresta plantaginifolia* | 14 | 0 |
| *Chresta pycnocephala* | 13 | 0 |
| *Chresta scapigera* | 1 | 1 |
| *Chresta souzae* | 5 | 0 |
| *Chresta speciosa* | 14 | 0 |
| *Chresta sphaerocephala* | 14 | 0 |
| *Chresta subverticillata* | 6 | 0 |
| *Chronopappus bifrons* | 0 | 0 |
| *Cyrtocymura scorpioides* | 8 | 0 |
| *Distephanus ambongensis* | 18 | 0 |
| *Elephantopus mollis* | 8 | 0 |
| *Elephantopus tomentosus* | 13 | 1 |
| *Eremanthus auriculatus* | 6 | 0 |
| *Eremanthus crotonoides* | 4 | 0 |
| *Eremanthus erythropappus* | 2 | 0 |
| *Eremanthus incanus* | 5 | 0 |
| *Gorceixia decurrens* | 12 | 0 |
| *Gymnanthemum amygdalinum* | 19 | 0 |
| *Heterocoma ekmaniana* | 2 | 0 |
| *Heterocypsela andersonii* | 12 | 0 |
| *Hololepis pedunculata* | 1 | 0 |
| *Lepidaploa opposita* | 14 | 0 |
| *Lessingianthus durus* | 8 | 0 |
| *Lessingianthus monocephalus* | 13 | 1 |
| *Lychnophora brunioides* | 3 | 0 |
| *Lychnophora granmogolensis* | 1 | 0 |
| *Lychnophora haplopappa* | 2 | 0 |
| *Lychnophora hatschbachii* | 6 | 0 |
| *Lychnophorella leucodendron* | 4 | 0 |
| *Lychnophorella morii* | 1 | 0 |
| *Lychnophorella santosii* | 0 | 0 |
| *Maschalostachys mellosilvae* | 0 | 0 |
| *Minasia pereirae* | 1 | 0 |
| *Moquinia racemosa* | 5 | 0 |
| *Munnozia gigantea* | 12 | 10 |
| *Paralychnophora harleyi* | 2 | 0 |
| *Piptolepis ericoides* | 2 | 0 |
| *Pseudostiffita kingii* | 4 | 0 |
| *Soaresia velutina* | 15 | 0 |
| *Stilpnopappus tomentosus* | 14 | 0 |
| *Stokesia laevis* | 13 | 5 |
| *Strophopappus speciosus* | 12 | 0 |
| *Vernonanthura patens* | 15 | 0 |
| *Vernonia gigantea* | 10 | 0 |
| *Vernonia missurica* | 6 | 0 |
| *Vernoniastrum ambiguum* | 12 | 0 |
